# Application of a Multiomics Imaging Workflow to Explore Asparlas Treatment in Solid Tumors

**DOI:** 10.1021/acs.analchem.5c01503

**Published:** 2025-06-11

**Authors:** Laura van der Vloet, Ronny Mohren, Christophe Bouillod, Ron M.A. Heeren, Michiel Vandenbosch, Pierre Barbier Saint Hilaire

**Affiliations:** † The Maastricht MultiModal Molecular Imaging (M4I) institute, Division of Imaging Mass Spectrometry (IMS), 5211 Maastricht University, Maastricht 6229 ER, Netherlands; ‡ Institut de Recherche et Développement SERVIER, 22 route 128, Gif-sur-Yvette, Paris-Saclay 91190, France

## Abstract

In acute lymphoblastic leukemia (ALL), hypermethylation of the asparagine synthetase (ASNS) gene promoter, leading to low levels of ASNS in tumor cells, is recognized as a prognostic biomarker, and l-asparaginase-based treatments (e.g., Asparlas) are frequently administered to these patients. In these cancers, tumor cells rely on external asparagine, and its depletion in the bloodstream results in tumor cell apoptosis. A multiomics (imaging) workflow is required to evaluate key molecular changes and characterize solid tumors to explore the potential efficacy of Asparlas in solid tumors. This study introduces a multiomics imaging workflow applicable to solid tumor specimens for the comprehensive molecular profiling of Asparlas treatment effects. The workflow integrates matrix-assisted laser desorption-ionization mass spectrometry imaging (MALDI-MSI), liquid chromatography coupled with high-resolution mass spectrometry, and histopathological staining on consecutive tumor tissue sections. It enables the detection and analysis of metabolites, lipids, and proteins. Tumor characterization was achieved through histology and clustering analysis based on lipid signatures, yielding consistent annotations. On-tissue chemical derivatization followed by MALDI-MSI was performed to assess metabolic alterations, with a focus on amino acids. ASNS distribution was mapped utilizing targeted MALDI-immunohistochemistry, followed by untargeted (spatial) proteomics on adjacent tissue sections. This study established a multiomics imaging approach and demonstrated its applicability in elucidating the metabolic changes in tumor tissue consequent to Asparlas treatment. Furthermore, it highlights the added value of multiomics imaging in pharmaceutical research and development.

## Graphical abstract

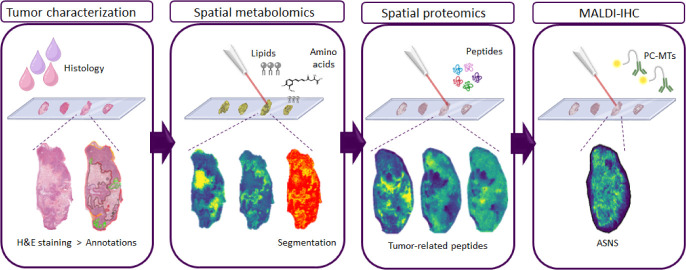

## Introduction

Cancer therapies have significantly progressed over the years. Tumor cells are characterized by distinct hallmarks differentiating them from healthy cells, including active proliferation, evasion of growth suppressors, resistance to cell death and senescence, high ability to induce angiogenesis, invasion, and metastatic potential, and phenotypic plasticity mainly linked to metabolic reprogramming. The latter allows tumor cells to increase nutrient absorption, supporting tumor survival, proliferation, and metastasis. One of the innovative therapeutic approaches involves exploiting this metabolic vulnerability by depriving tumor cells of amino acids.^1^ Some tumor cells are highly dependent on amino acids, such as the nonessential amino acid asparagine (Asn). Asn is crucial for cell maintenance and growth, and, particularly in tumors, for promoting cell survival and proliferation.^2^ Asn is synthesized by the enzyme asparagine synthetase (ASNS) in an ATP-dependent manner through a glutamine (Gln)-amino group transamination reaction to aspartic acid (Asp). Elevated ASNS expression promotes metastatic progression and is a marker of poor prognosis in some tumor types.^3^ Therefore, understanding the role of Asn and ASNS activity in solid tumors may open new opportunities for personalized cancer therapies.

For several decades, l-asparaginase (L-ASNase) has been clinically used as a therapeutic treatment against acute lymphoblastic leukemia (ALL) in infants and young adults. L-ASNase is a homotetrameric amidohydrolase enzyme that depletes Asn by converting it into Asp and ammonia. This depletion strategy selectively targets tumor cells lacking ASNS, as they cannot synthesize Asn *de novo* due to the hypermethylation of the ASNS gene promoter, while sparing healthy cells capable of Asn synthesis.^4^ Calaspargase pegol (Asparlas, SERVIER), an FDA-approved drug, consists of E. coli L-ASNase and is PEGylated to extend its half-life.^5^ Emerging evidence suggests that L-ASNase-based treatments could benefit other subtypes of leukemia or aggressive solid tumors.^6,7^ The potential repurposing of Asparlas for solid tumors highlights an exciting frontier in oncology, where metabolic therapies can complement existing approaches to target specific vulnerabilities in tumor cells. Despite the potential of Asparlas for treating solid tumors, several challenges remain. Unlike hematologic tumors, solid tumors present a heterogeneous environment where nutrient supply, metabolic demands, and microenvironments vary widely. This potentially results in an inconsistent Asn depletion. Additionally, ASNS expression could help predicting the efficacy of Asparlas treatment, allowing for a more personalized therapeutic approach.^3^ There are two factors that can be linked to the ineffectiveness of L-ASNase based treatment, including toxicity and resistance mechanisms. Toxicity affects the efficacy of L-ASNase-based treatment mainly due to severe immunological side effects, coagulopathy, liver toxicities, and glutaminase coactivity.^2^

Amino acids are fundamental to various metabolic processes, as their disorders are closely associated with various diseases. For example, amino acid concentrations and distributions significantly differ between healthy and tumor cells. Therefore, it is essential to perform spatial characterization of amino acids in solid tumors to elucidate their roles. Matrix-assisted laser desorption/ionization mass spectrometry imaging (MALDI-MSI) is an analytical technique that enables the simultaneous visualization of exogenous and endogenous molecules in a label-free manner *in situ*. Amino acids can be detected, and the distribution can be mapped, with enhanced sensitivity by performing on tissue chemical derivatization (OTCD) prior to analysis.^8,9^ OTCD improves ionization efficiency, facilitating the detection of low abundant molecules. Amine groups present in amino acids can be targeted by OTCD compounds to enhance detection sensitivity.^9^ In oncology, it is crucial to analyze the acquired MSI data by taking into consideration the histological information. Hematoxylin and eosin (H&E) staining followed by annotations of a trained pathologist is considered to be the golden standard to analyze tissue morphology.^10^ More recently, MALDI-MSI has increasingly been used to differentiate between different tumor regions within a single section.^10–12^ Typically, *in situ* spatial lipidomic analysis has been used to segment the tissue based on molecular footprint clustering. The lipid signals in the clusters provide insights into the functional lipidomic organization within the tumor, often aligning with histopathological staining.^10,12^

Cancer is characterized by heterogeneity and a unique and complex tumor microenvironment. Proteins are key players in major processes in healthy and tumor tissues, and alterations in protein regulation have been seen as crucial in tumor progression. Spatial information in tumor tissues is lost in most bulk proteomic analyses.^13^ The visualization of intact proteins using MALDI-MSI remains limited due to insufficient mass resolution and poor sensitivity for low abundant proteins. Untargeted on-tissue bottom-up spatial proteomics, where on-tissue digestion is performed prior to MALDI-MSI is therefore suggested as an alternative for analyzing protein distribution in tissue. However, matching peptides to their corresponding proteins is still challenging. Protein identification requires tandem MS/MS fragmentation or ultrahigh mass resolution instruments.^14^ A novel strategy to overcome this limitation is MALDI-immunohistochemistry (IHC), a targeted antibody imaging approach based on the detection of photocleavable mass-tags (PC-MTs). PC-MTs are modified polypeptides that are linked to the antibodies of interest, which can then be detected and visualized using MALDI-MSI in a multiplexed fashion.^15^ This approach enables label-free untargeted MSI, followed by a labeled PC-MT-based targeted MSI of macromolecular biomarkers of interest, providing multiple omics datasets from the same tissue slide.^15,16^

In this study, we developed a multiomics workflow that integrates MALDI-MSI of lipids, metabolites, and proteins in combination with tandem liquid chromatography mass spectrometry (LC-MS/MS) on fresh frozen SNU-601 induced tumor sections to study Asparlas efficacy. To evaluate the tissue morphology, these tissue sections were stained with classical histology staining, followed by annotating the tumor tissue. We established a multimodal imaging workflow and demonstrated its added value for enhanced assessment and understanding of the metabolic changes occurring in solid tumor tissue after Asparlas treatment.

## Methods and Materials

### Chemicals and Reagents

4-Hydroxy-3-methoxycinnamaldehyde (CA), α-cyano-4-hydroxycinnamic acid (CHCA), 2,5-dihydroxybenzoic acid (DHB), methanol (MeOH; ULC-MS grade), ethanol (EtOH; ULC-MS grade), HPLC grade water, acetonitrile (ACN), and chloroform (≥99%) were purchased from Fisher Scientific (Loughborough, Leicestershire, U.K.). Ammonium bicarbonate (ABC), dithiothreitol (DDT), eosin-Y (Avantor), formic acid (FA, ULC grade), Gill’s hematoxylin, iodoacetamide (IAM), norharmane, trifluoroacetic acid (TFA, ULC grade), xylene, paraformaldehyde (PFA), phosphate buffered saline (PBS 1×), and methyl-tert butyl ether (MTBE) were purchased from Sigma-Aldrich (Zwijndrecht, The Netherlands). Entellan was purchased from Merck (Burlington, MA). RapiGest SF was purchased from Waters (Milford, USA). Trypsin/LysC and trypsin were obtained from Promega (Madison, USA).

### Animal Experiments

The generation of animal samples was strictly reviewed and approved by the Institutional Animal Care and Use Committee (IACUC) of Crown Bioscience and SERVIER and was conducted in accordance with the regulations of the Association for Assessment and Accreditation of Laboratory Animal Care (AAALAC). SNU-601 tumor was induced by injecting cells (1 × 10^7^) subcutaneously at the right front flank region (female BALB/c nude mice) in 0.2 mL of PBS mixed with Matrigel (1:1) for tumor development. Once the tumor size reached an area of 100–200 mm^3^, Asparlas treatments were administered intravenously (iv) every 4 days up to day 28 using a dose level of 20 IU/kg. At the end of the experiment, mice were sacrificed, and tumor samples were snap frozen for downstream analysis. Mice were sacrificed 4 days after the seventh Asparlas dosage (*N* = 2) and 2 h after the eighth Asparlas dosage (*N* = 2).

### Tissue Sectioning

SNU-601 induced tumor tissues were sectioned at 12 μm using a cryotome (Leica, Rijswijk, The Netherlands) at −20 °C, and thaw-mounted onto indium–tin oxide coated glass slides (ITO, CG-40IN-S115, Delta Technologies, USA). Slides were stored at −80 °C until further use.

### On-Tissue Derivatization and Matrix Application for Spatial Metabolomics and Lipidomics

CA on-tissue derivatization was applied to tumor tissue sections prior to matrix application. Briefly, fresh frozen sections were dried in a desiccator for 15 min. A CA derivatization solution was prepared by dissolving 4 mg/mL in 70% MeOH. The CA solution was sprayed with an HTX TM sprayer (HTX Technologies, Chapel Hill, NC). Spraying parameters were as follows: temperature; 60 °C, nozzle velocity; 600 mm/min, flow rate; 0.10 mL/min, number of passes; 2, track spacing; 2 mm, and nitrogen gas pressure of 10 psi. The slide was put in a humid incubation chamber at 37 °C for 16 h. DHB matrix (15 mg/mL in 70 MeOH + 0.2% TFA) was applied on tissue using the HTX TM sprayer (HTX Technologies LLC, Carrboro) using the following settings: temperature; 60 °C ; nozzle velocity; 1100 mm/min, flow rate; 0.10 mL/min, number of passes; 6, track spacing; 2 mm, and nitrogen gas pressure of 10 psi.

### Photocleavable Mass-Tag Labeling of Asparagine Synthetase Antibody

A commercially available anti-ASNS antibody (Proteintech, 14681-1-AP) was labeled with a novel PC-MT (*m*/*z* 1506.77; AmberGen, Inc.), followed by tissue staining and MALDI-MSI analysis. The confirmation of the PC-MT labeling is shown in Figure S5. Briefly, to preclean the unlabeled ASNS antibody, 100 μg was added into a spin filter and centrifuged for 5 min at 13,500 × *g*. The flow-through was discarded, and antibody buffer was added to the spin filter and centrifuged for 10 min at 13,500 × *g*. The cleaned antibody was collected in 80 μL of antibody buffer by inverting the spin filter into a new microcentrifuge tube, followed by centrifugation for 2 min at 800 × *g*. Next, the precleaned ASNS antibody was labeled with the PC-MT by first adding reaction buffer to the ASNS antibody, followed by gently vortexing for 5 s. Consecutively, single-use PC-MT solution was added to the antibody solution that was followed by gently vortexing for 5 s. The antibody solution was incubated for 1 h at RT at 500 rpm. This was followed by the addition of a quencher solution to the antibody solution, which was allowed to react for 15 min at RT and 500 rpm. The PC-MT labeled ASNS antibody was purified by transferring the antibody solution into a spin filter, which was then centrifuged for 5 min at 13,500 × *g*. The flow-through was discarded, and the spin filter was washed five times by adding washing buffer to the spin filter and centrifuging for 5 min at 13,500 × *g*. The purified antibody was collected in 70 μL washing buffer by inverting the spin filter into a new microcentrifuge tube, which was centrifuged for 2 min at 800 × *g*. Lastly, storage buffer was added to the PC-MT labeled antibody and stored at −20 °Cuntil further use. Photocleavable reagents were protected from light during all incubation steps and during storage, but they were handled under ambient laboratory lighting.

### MALDI-IHC of PC-MT Labeled ASNS

Fresh frozen SNU-601 induced tumor tissues were stained with the PC-MT labeled ASNS antibody using a protocol previously reported.^15^ Briefly, the Miralys probes were photocleaved by the illumination of UV light at 365 nm with a Phrozen UV curing lamp for 5 min (3 mW/cm^2^) to achieve maximum photocleavage. Matrix was then sublimed on the stained sections using 50 mg DHB in acetone at 160 °̊C for 180 s (HTX Sublimator, HTX Technologies, Chapel Hill, NC). The matrix was recrystallized in an oven at 50 °̊C for 90 s using a preheated Petri dish with 0.5% EtOH in water.

### On-Tissue Protein Digestion Followed by Peptide MALDI-MSI

Fresh frozen SNU-601 induced tumor tissue was first washed and fixed by immersing the slides twice in ice-cold 100% EtOH for 2 min, once in 96% EtOH for 1 min, once in 70% EtOH for 1 min, and twice in ice-cold HPLC grade water for 2 min. The slides were dried in a desiccator, and trypsin was freshly prepared by adding 200 μL of cold HPLC-grade water to 20 μg of trypsin. Trypsin was sprayed with an HTX M3+ sprayer (HTX Technologies LLC, Carrboro). Spraying parameters were as follows: temperature; 45 °C, nozzle velocity; 1200 mm/min, flow rate; 30 μL/min, number of passes; 8, track spacing; 2.5 mm, and nitrogen gas pressure of 10 psi. The slide was put in an incubation chamber at 37 °C for 16 h. CHCA matrix solution (10 mg/mL in 70% ACN + 1% TFA) was applied with the HTX M3+ sprayer (HTX Technologies LLC, Carrboro) using the following parameters: temperature; 75 °C, nozzle velocity; 1200 mm/min, flow rate; 120 μL/min, number of passes; 4, track spacing; 1.5 mm, and nitrogen gas pressure of 10 psi. After being sprayed, slides were dipped in ice-cold 100 mM ammonium phosphate monobasic solution and dried vertically in a desiccator.

### Data Analysis and Peptide Identification

Peptides found in the MALDI-MSI dataset were analyzed as follows: principal component analysis (PCA) was performed using the following parameters: no denoising or Pareto scaling and with 10 components. Discriminative analysis was executed by performing a receiver operating characteristic (ROC) using an area under the curve (AUC) threshold of 0.7 and 0.3 for significance. These peaks were then matched with the LC-MS/MS data. An *m*/*z* value was accepted to be identified as peptides, if at least three peptides correlating to the same protein were found in the MALDI-MSI data set that had similar intensities and thereby showing identical distribution within the tissue samples.

### MALDI-MSI and Data Analysis

All MALDI-MSI data were acquired on a timsTOF fleX instrument (Bruker Daltonics GmbH, Germany) in positive ionization mode at a pixel size of 50 × 50 μm. The method was externally calibrated by using red phosphorus before the imaging experiment. FlexImaging version 5.0 (Bruker Daltonics GmbH, Bremen, Germany) and SCiLS lab 2025a (SCiLS GmbH, Bremen, Germany) were used for processing and analyzing the data. Data were normalized to the RMS and mean spectra was analyzed. Peak picking was performed in mMass (version 5.5.0) using the following parameters: signal-to-noise ratio threshold 3, absolute intensity 0, relative intensity 0, picking height 50, baseline, smoothing, and deisotoping peaks were enabled. Significant differences in metabolic profile between the control and Asparlas treated group was determined by performing a one-way ANOVA on the RMS normalized MALDI-MSI data set. Metabolites were considered significantly altered when the *p*-value was <0.05. To control for multiple hypothesis testing, a Benjamini-Hochberg False Discovery Rate (FDR) correction at a threshold of *q* < 0.05.

### Histological Staining

On consecutive slides, a standard protocol was used to counterstain for measured MALDI-MSI slides with hematoxylin and eosin (H&E, Merck KGaA, Darmstadt, Germany). In the Supplementary Material and Methods and Materials a step by step protocol is presented. The Aperio CS2 scanner (Leica Microsystems) was used for whole slide scanning and digitalization at a 20× magnification.

## Results and Discussion

### SNU-601 Tumor Characterization via Histology and Molecular Profiling

A typical example of multimodal imaging is registering MSI with histology, linking the distribution of molecules to the interfaces between viable and necrotic tumor regions.^17^ The molecular imaging technique MALDI-MSI complements histopathological analysis and enables the simultaneous analysis of hundreds of molecular compounds in a single measurement. MALDI-MSI has been implemented to characterize solid gastric tumors via spatial lipidomics profiling, which is essential for understanding the cellular physiology and pathology of various cancer types.^12^ In this study, spatial lipidomic analysis was implemented to further examine molecular footprint clustering in addition to classical histology. Molecular footprint clustering is an unbiased approach to annotate biological tissue specimens, while the quality of H&E-based annotations strongly depends on the scientist/pathologist. In this study, spatial lipidomics based segmentation was used to complement classical H&E-based annotations. A MALDI-MSI workflow was optimized to simultaneously map the metabolome and lipidome. OTCD was performed to improve the sensitivity and specificity of small metabolites (e.g., amino acids). CA was used as derivatization reagent, followed by MALDI-MSI analysis. The average spectrum obtained with MALDI-MSI, containing spatial metabolic and lipidomic data, is presented in Figure S1. Since the CA derivatization stained the tissue yellow/green, a consecutive slide was required for histology (Figure 1). Tumor regions were annotated as followed: regions surrounded in red were classified as tumor tissue, the black surrounded regions were classified as necrotic regions, and all other colors were categorized as stroma cells (e.g., immune cells and blood vessels). Control and Asparlas dosed tissues were assessed based on the H&E staining and molecular clustering (Figure S2).

**Figure 1:**
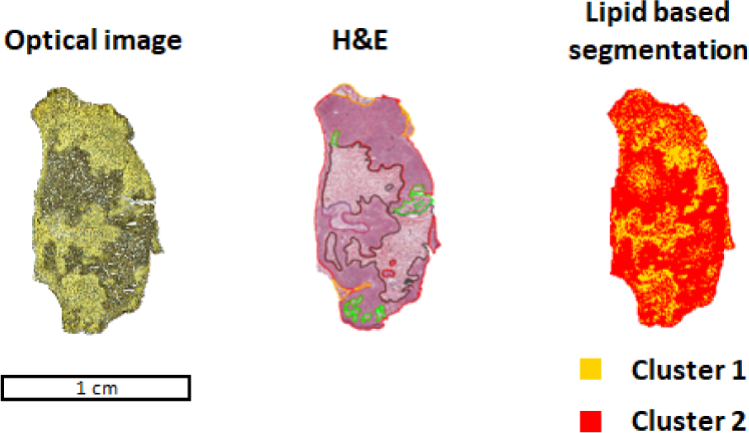
SNU-601 induced tumor characterization via histology and molecular footprint clustering. CA derivatization was applied prior MALDI-MSI analysis to enable the simultaneous detection of metabolites (e.g., amino acids) and lipids. CA derivatization caused a yellow stain on tissue, for which histology was performed on a consecutive slide, followed by annotations. H&E-based annotations were categorized in red = tumor, black = necrosis, green = exudate, blue = stroma, yellow = immune cells. Based on acquired spatial lipidomic data, segmentation was performed, which resulted in two main clusters. Cluster 1 in yellow represents tumor tissue, and cluster 2 in red represents necrotic and stroma tissue.

The acquired lipidomic data were then used to generate a segmentation map (Figure 1). Two main clusters could be distinguished from each other. Cluster 1 in yellow in the segmentation map denotes tumor tissue, whereas cluster 2 in red indicates necrotic regions. Figure 2A presents the average spectrum obtained from the MALDI-MSI analysis, zoomed in at the lipid range. The measured *m*/*z* values were matched to possible lipid entities using the LIPID MAPS database. A total of 59 lipids were fully identified in the MALDI-MSI data set and used for clustering (Table 1). ROC analysis was used to analyze the importance of each lipid in cluster separation. Only 12 lipids were mostly accountable for the separation of the two clusters (Figure 2B,C). Those 12 lipids were most abundant in the tumor regions of the tissue (Figure 2). An overview of all measured control and Asparlas dosed tissues is presented in Figure S3. Those lipids are highlighted in a volcano plot, in which the absolute ROC values are plotted against the log_2_ fold change of the average peak area. PCs were mainly responsible for the cluster separation, and correlated ion images of the 12 lipids are presented in Figure 2C. A total of 299 lipids were detected in LC-HRMS analysis. Peaks were matched to the monoisotopic molecular masses of lipids that contained H^+^ or Na^+^ adducts. The 59 lipids from the MALDI-MSI data set were identified using the LC-HRMS data set and are presented in Table S1. Phosphatidylcholines (PCs) and phosphatidylethanolamines (PEs) were the most abundant phospholipids. In line with our findings, glycerophospholipids and glycerolipids are also known as the major lipid classes found in human head and neck tumors.^12^

**Table 1:** Identified Lipid Species in SNU-601 Induced Tumor Tissue

Lipid class	Abbreviation	Amount of identified lipids
Lysophosphatidylcholine	LPC	4
Ceramide	Cer	10
Sphingomyelin	SM	4
Phosphatidylcholine	PC	29
Phosphatidylethanolamine	PE	12

**Figure 2:**
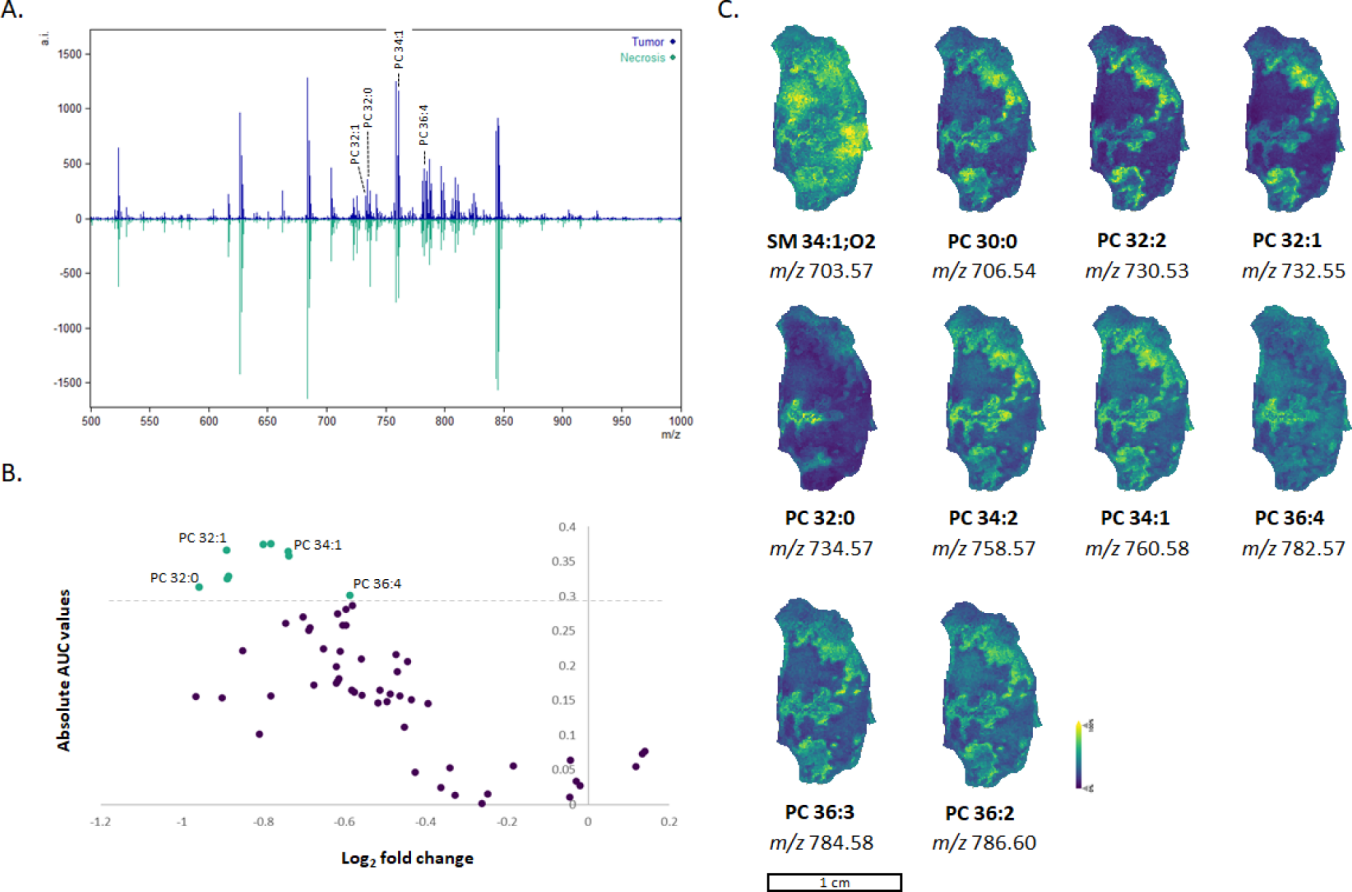
Spatial lipidomic analysis of SNU-601 induced tumor tissue. **A.** Average spectrum of the obtained MALDI-MSI analysis. In blue: average spectrum of the tumor regions. In green: average spectrum of the necrotic regions. **B.** Volcano plot of the 59 identified lipids from the MALDI-MSI data set. In green are the lipids that were found to be significantly upregulated in tumor regions compared to the necrotic regions. **C.** Ion images of the 10 lipids that are primarily responsible for the segmentation clustering.

When comparing histology with the segmentation map generated with MALDI-MSI, we observed that more regions could be distinguished from each other via histology, followed by manual annotations. In routine clinical practice, pathologists base their histological diagnoses on visual recognition, semiquantification, and integration of multiple morphological features of the analyzed samples. Histopathology analysis is inherently limited by its subjective nature and the natural differences in visual perception, data integration, and judgment between independent observers.^18^ Recently, advances in artificial intelligence (AI) have been implied in digital pathology, altering the way cancer can be diagnosed and classified. AI has been applied to a variety of image processing and classification tasks, including low-level tasks (e.g., segmentation) and higher-level tasks (e.g., disease diagnosis prediction and treatment prognosis based on patterns in the image). AI tools can provide a unique platform for innovations and advances in anatomical and clinical pathology workflows, especially when diagnostic algorithms are incorporated.^18,19^ Molecular footprint clustering corresponds to the composition of the analyzed tissue, highlighting the molecular similarity of morphological structures. Segmentation can therefore lead to improved understanding of functional processes in tissue.^20^ Molecular clustering depends on the number of pixels included in the data analysis, which can significantly influence the quality of the lipid-based segmentation. Machine learning and clustering algorithms used in segmentation (e.g., k-means, hierarchical clustering) are more reliable when trained on a large data set. A limited sample size might lead to inferior separation of lipid regions.^21^ MALDI-MSI experiments generate hundreds of molecular images in a single run, adding complexity to the interpretation of data sets.

### Spatial Metabolomics of SNU-601 Induced Tumor Tissue to Investigate Asn and Gln Distribution

After characterizing the distinct regions within the solid gastric tumors, the aim was to specifically detect Asn and Gln in these tumors to enable spatial monitoring of the pharmacodynamic effect of Asparlas treatment. As previously mentioned, free amino acids such as Asn are not detectable without derivatization. First, a method was optimized to simultaneously map the metabolome and lipidome using MALDI-MSI. In this work, CA-OTCD prior to MALDI-MSI analysis was necessary to enhance the intensity of amino acids (such as Asn and Gln) within the solid gastric tumor tissues. Other commonly used OTCD reagents that target the amine group of molecules include *p*-*N*,*N*,*N*-trimethylammonioanilyl *N*′-hydroxysuccinimidyl carbamate iodide (TAHS) and 2,4-diphenyl-pyranilium tetrafluoroborate (DPP-TFB).^22^ CA derivatization was demonstrated to provide the highest intensity of certain amino acids in the brain, including glutamine, glutamate, and aspartate, which are amino acids that are involved in the biosynthesis of Asn. A representative reaction between CA and Asn is shown in Figure 3A. The functional aldehyde group of the CA molecule reacts easily with primary amines, forming a stable Schiff’s base.^23,24^ Optimizing OTCD requires balancing reagent concentration, solvent composition, reagent solubility, and reaction time to ensure reproducibility, prevent analyte delocalization, and preserve tissue integrity under mild conditions.^9^

**Figure 3:**
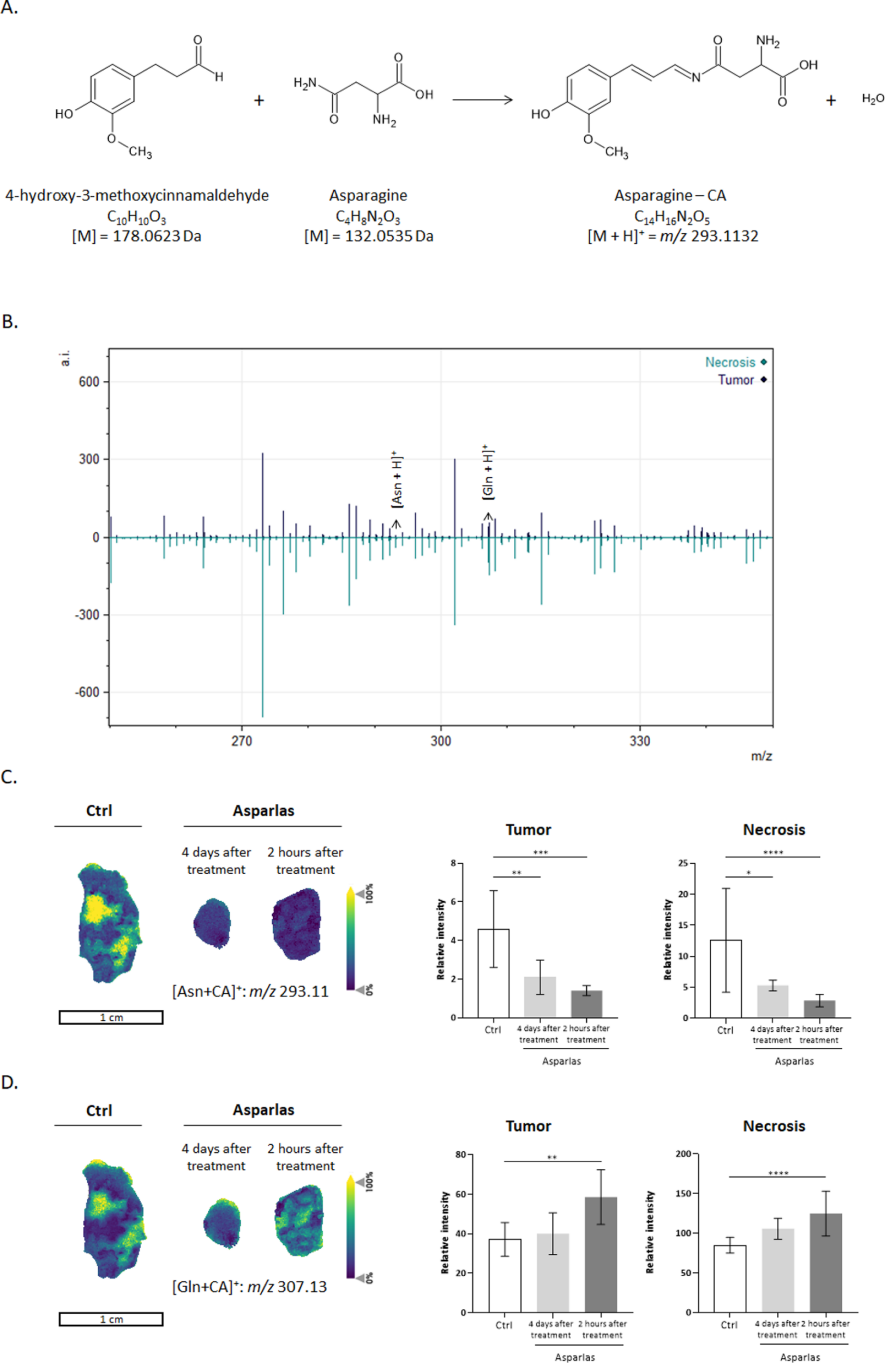
Metabolic profiling of Asparlas dosed SNU-601 induced tumor tissue after performing OTCD and MALDI-MSI. **A.** Derivatization reaction between 4-hydroxy-3-methoxycinnamaldehyde (CA) and asparagine (Asn). **B.** Average spectrum of MALDI-MSI generated analysis in the CA-derivatized amino acid region after root-mean-square (RMS) normalization. **C.** and **D.** MALDI-MSI analysis of amino acids, including Asn and glutamine (Gln) in SNU-601 induced tumor tissue. The distribution of CA-Asn (*m*/*z* 293)^11^ and of CA-Gln (*m*/*z* 307)^13^ was visualized after RMS normalization. The bars represent RMS normalized intensities of Asn and Gln in tumor and necrotic regions. Significance was determined by performing an ANOVA on the RMS normalized relative intensities of Asn and Gln. Significance is shown as *p < 0.05, **p < 0.01, ***p < 0.001, and ****p < 0.0001.

Annotated necrotic and tumor regions were delineated in SCiLS Lab, and detected derivatized amino acids were subsequently analyzed per region. Data normalization was performed using the Root Mean Square (RMS) method. The average spectrum of the derivatized amino acid in tumor and necrotic regions is presented in Figure 3.A difference in Asn normalized signal was observable within tumors and across treatment conditions. Asn is exhibited to have a higher level in necrotic regions compared to tumor regions. Necrotic regions are often characterized by hypoxia and nutrient deprivation.^25^ Under such conditions, nonhypermethylated cells may upregulate ASNS to synthesize Asn, potentially increasing Asn levels to support tumor progression.^26^ In the few analyzed samples, a depletion of Asn was observed in necrotic and tumor regions following Asparlas treatment (Figure 3C,D). An overview of all measured control and Asparlas dosed tumor tissues is presented in Figure S4.

This proposed multiomics approach allows for the analysis of several additional amino acids, including Gln. Gln is of particular interest due to the observed glutaminase coactivity of some asparaginase-based treatments.^27^ Gln demonstrates a higher signal in necrotic regions compared to tumor regions. Following Asparlas treatment, an increase in Gln levels was observed in necrotic and tumor regions. As Gln metabolism is essential for cancer cell survival and proliferation, ASNase-based treatments with low glutaminase coactivity may result in lower toxicity and prolonged treatment.^3,27^

The spatial metabolomic workflow used in this study provides additional spatial insights into metabolic processes that are tumor region specific. Asparlas is used to deplete Asn in ALL patients, and our hypothesis was that Asparlas can be used for the same purpose in solid tumor models. Spatial metabolomics analysis is used to assess Asn levels per tumor region. Since Asparlas treatment is not effective in all tumor types (depending if the tumor is asparaginase-resistant), utilizing MALDI-MSI, a direct correlation can be observed whether Asn is decreased in specific regions of the tumor sample (e.g., cell dependent) or an overall reduction of Asn in the tumor sample post-Asparlas treatment. Using LC-MS/MS analysis only, you can also assess amino acid levels but the spatial information will be lost. However, additional metabolomic tandem LC-MS analysis would allow for the quantitative analysis of amino acids of interest. Applying an internal standard prior MALDI-MSI analysis would enable the absolute quantification of amino acids per region of interest (ROI).^11^

### Untargeted (Spatial) Proteomics Analysis for In-Depth Molecular Pathway Analysis

Since Asparlas targets Asn, an amino acid crucial in protein translation, we explored the proteome by combining spatial proteomics analysis with LC-HRMS based label free proteomics experiments. LC-HRMS based proteomics analysis lacks spatial information but provides a more comprehensive overview of protein metabolism within tissue and is used to match peptide peaks from the MALDI-MSI data set with their corresponding protein ID. The protein identification method used is based on previously published articles that combined spatial proteomics with tandem LC-HRMS.^13,14^ Peptide masses were matched with accurate peptides from the LC-HRMS data set. At least three peptide sequences and masses (LC-HRMS data set) must match with MALDI-MSI peptide masses, providing similar peptide distributions (Table S2).

First, untargeted spatial proteomics was performed after on-tissue protein digestion to map peptide distribution throughout the tumor tissue samples, providing information on protein metabolism in specific regions of the tumor (Figure 4B). A total of 116 peptides were mapped. These peptides were used for ROC analysis to define significantly altered peptides in solid tumors after Asparlas treatment. All significantly altered peptides are presented in Tables S3 and S4. To match those peptides with their corresponding proteins, bottom-up LC-HRMS proteomics was used. A total of 7457 peptides corresponding to 1974 proteins were detected. Combining MALDI-MSI and LC-HRMS data set led to the identification of 24 spatially resolved proteins. Ion images of typical tumor markers, such as collagen-α1, actin, myosin, and keratin-18, were reduced after Asparlas treatment (Figure 4B). Respectively, they are used as markers for the extracellular matrix, cytoskeleton, muscle differentiation, and epithelial origin. Collagen is a major component of the tumor microenvironment and plays a crucial role in cancer fibrosis.^28^ Keratin-18 is a marker of apoptosis and is associated with tumor progression.^29^ This suggests that Asparlas probably affects apoptosis or differentiation pathways and that changes in the tumor microenvironment occur. PCA was then performed to observe potential group clustering (Figure 4A). For the MALDI-MSI data set, matched peptides were used to test for group clustering. Separation of the control and Asparlas-treated groups was observed, which was mainly caused by PC_3_. PCA was also performed on the 1974 detected proteins (FDR < 1%) from the LC-HRMS (Figure S5), which also showed the separation of the control group with the two Asparlas treated groups. All detected proteins are presented in a volcano plot (Figure S6), in green, presenting the significantly altered proteins. Using MALDI-MSI to map the spatial distribution of peptides highlights specific regions within the tissue that are affected by Asparlas. This provides information if the drug is effective in the tumor itself or also has some off target effects. When combining MALDI-MSI with laser microdissection based on identified clusters or histology annotations, followed by LC-HRMS allows for in-depth molecular analysis. This multimodal approach highlights its added value in the analysis of novel therapeutic drugs in pharmaceutical research and development, providing enhanced understanding of metabolic processes in specific tumor regions.

**Figure 4:**
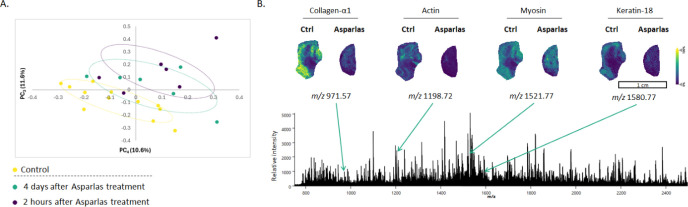
Untargeted (spatial) proteomics analysis of SNU-601 induced tumor tissue. **A.** A 2D PCA plot of the spatial proteomics MALDI-MSI data set, with the axes representing PC_3_ and PC_4_. 95% confidence ellipses are presented for each experimental group. **B.** Representative peptide spectrum after root-mean-square (RMS) normalization of SNU-601 induced tumor tissue measured by MALDI-MSI. The spatial distribution of four features that were identified as peptides is presented: collagen-α1 (*m*/*z* 971.57), actin (*m*/*z* 1198.72), myosin (*m*/*z* 1521.77), and keratine-18 (*m*/*z* 1580.77).

### ASNS Distribution in SNU-601 Induced Tumor Tissue Using MALDI-IHC

Spatial proteomics analysis lacks sensitivity to detect low abundant analytes or analytes that are difficult to ionize. ASNS catalyzes the synthesis of Asn and Glu from Asp and Gln in an ATP-dependent amidotransferase reaction (Figure 5A). As previously discussed, Asn depletion through the Asparlas treatment might induce ASNS as a compensatory mechanism. Enhanced ASNS activity results in promoted cell proliferation, chemoresistance, and metastatic behavior.^30^ ASNS expression levels are believed to be a key factor in resistance mechanisms to L-ASNase based treatments.^31^ In addition, some tumor cell lines exhibit low ASNS native expression due to hypermethylation of the gene promoter. Being able to assess ASNS distribution within tumors to evaluate changes in its expression could provide insights into potential resistance mechanisms in future studies. In this work, a targeted MALDI-MSI approach, termed MALDI-IHC, was used to localize ASNS in tumor tissue (Figure 5). During the MALDI-IHC sample preparation, lipids are removed from the tissue to enhance sensitivity of the PC-MT labeled antibody by decreasing ion suppression. For classical IHC protocols, antibodies must exhibit high specificity for their target to produce reliable antibody detection. Using MALDI-IHC, a minimal amount of antibody (100 μg) is required during the labeling process. Too low final antibody concentrations can lead to unsuccessful data acquisition. Thereby, antibodies stored in high glycerol levels are not validated for MALDI-IHC purposes, as it can lower the final labeled concentration of the antibody. In this work, a commercially available ASNS antibody was labeled with a novel PC-MT probe. The anti-ASNS antibody was validated performing classical IHC staining. As a positive control, cell lines Ocily-3 and RPMI-8226 were used. RS4-11 was used as a negative control (Figure S6). Successful antibody labeling was confirmed prior to staining the gastric tumor tissues (Figure S7). ASNS was distributed throughout the whole tissue section (Figure 5). No alterations were observed following Asparlas treatment. In most healthy and tumor cells, *ASNS* levels increase rapidly after Asn depletion. However, protein synthesis is not increased in the same rate,^2^ suggesting that Asparlas may not have an immediate effect on ASNS protein expression and activity.

**Figure 5:**
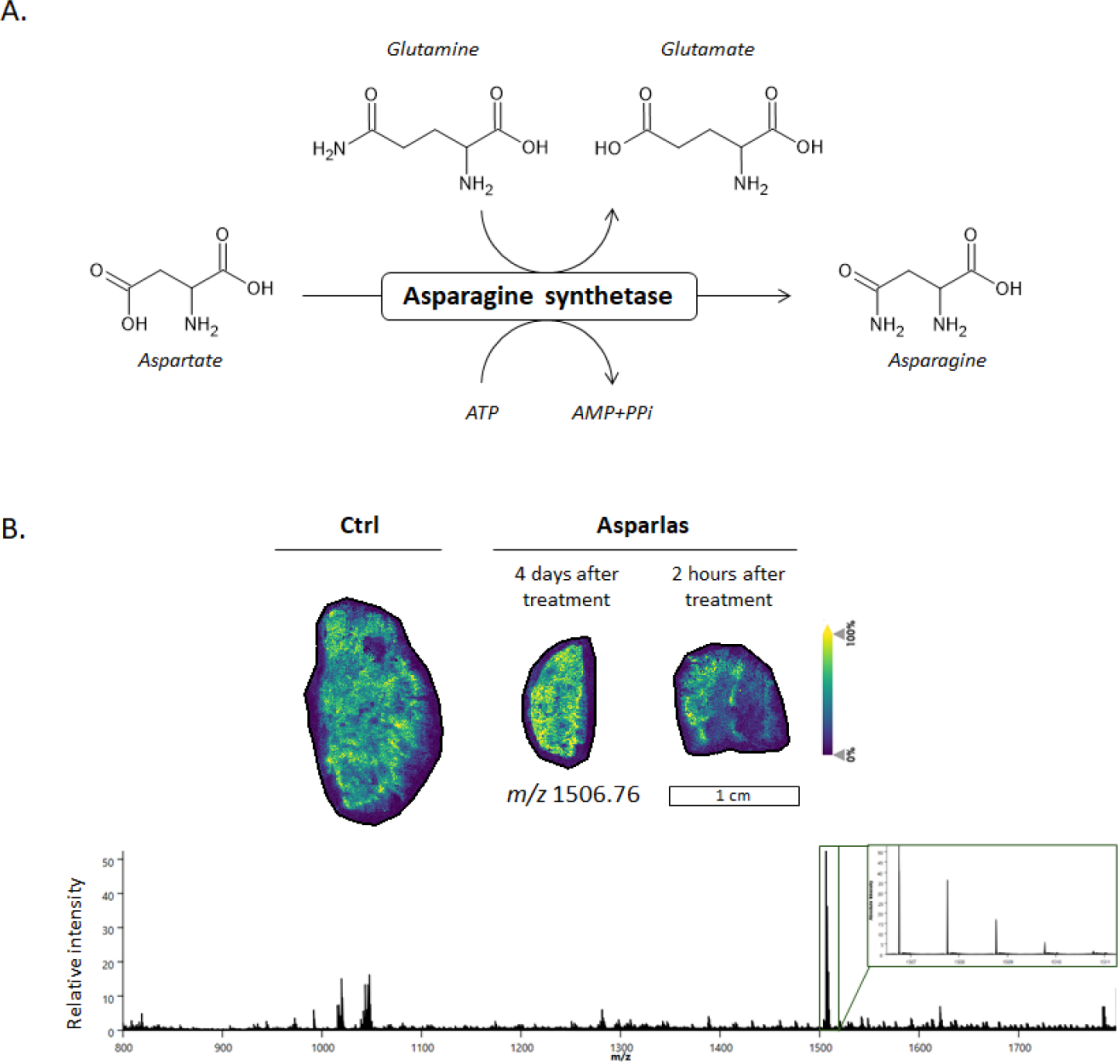
Asparagine synthesis (ASNS) distribution in SNU-601 induced tumor tissue using MALDI-IHC. **A.** Asparagine is synthesized in an ATP-dependent manner from aspartate and glutamine by asparagine synthetase. **B.** MALDI-IHC was performed using a PCMT-labeled ASNS antibody (label *m*/*z* 1506.77), and the corresponding ion images is presented. Average spectrum of the control sample is shown using a root-mean-square (RMS) normalization.

MALDI-IHC approaches allow for single-cell resolution and less delocalization compared to on-tissue digestion methods. MALDI-IHC enables multiplexing, providing complementary data from the same tissue section and facilitating straightforward visualization and identification of proteins.^15^ Advantages of MALDI-IHC over classical histology staining include the subsequent imaging of lipids, followed by MALDI-IHC MSI on the same tissue section. ROIs can then be dissected and further analyzed by tandem LC-MS.^16^ Commercially available tumor-dedicated antibody panels, with the addition of antibodies of interest, can be used in a single multiplex MALDI-IHC MSI experiment. This approach prevents signal overlap and enables higher multiplexing compared with classical histology staining. This allows for the simultaneous characterization of the tumor and the investigation of biomarkers related to disease progression or treatment. Besides targeting biomarkers of interest, it is also possible to label the therapeutic drug candidate with a PC-MT label, allowing simultaneous mapping of the distribution of the drug and proteins that provide insights into morphology, involved molecular pathways, and drug efficacy in specific regions. While MALDI-IHC offers significant advantages over classical immunohistochemistry staining, including the ability to overcome multiplexing limitations,^15,16,32^ it requires expensive equipment and expertise. However, MALDI HiPLEX-IHC is a powerful tool for research and diagnostic applications, providing a deeper understanding of complex molecular landscapes in tissue samples.

## Conclusion

This study presents an enhanced multiomics imaging approach to study Asparlas as a potential treatment for solid tumors. Over the years, significant progress has been made in applying MSI to study biomolecular distributions in heterogeneous tumors. Many biologically relevant lipids, metabolites, and peptides are detectable by MALDI-MSI. Lipids and amino acids within Asparlas treated tumor tissue samples are simultaneously mapped in a single MALDI-MSI run. The *in situ* spatial lipidomic data set was used to segment and characterize tumor tissue. Classical histology staining was performed on a consecutive slide to compare the reliability of the segmentation with manually performed annotations validated by a trained pathologist. Our data indicate that Asn is depleted in tumor tissue as a consequence of Asparlas treatment. Untargeted spatial proteomics was performed to assess affected proteins in specific tumor regions. A targeted MALDI-IHC-MSI approach was required to visualize the ASNS. ASNS levels did not seem to be affected by the Asparlas treatment. Tandem LC-MS was performed for both lipidomic and proteomic analyses, allowing us to identify the lipids and match the peptides with their corresponding proteins. The low number of mice used per experimental group (*N* = 2) in this work limits the conclusions made on the biological impact of Asparlas treatment. However, the proteomics analysis indicates alterations in typical tumor biomarkers. In conclusion, we established a multiomics imaging workflow and demonstrated its added value in enhancing the assessment and understanding of metabolic changes occurring in tumor tissue after Asparlas treatment. These multiomics imaging approaches demonstrated clear added value in pharmaceutical research and development.

## Supplementary Material



## Notes

Supplementary methods and materials; lipid extraction of whole tumor tissue; LC-MS/MS lipidomics analysis; MALDI-IHC sample preparation; PC-MT labeling confirmation; proteomics sample preparation; LC-MS/MS proteomics analysis and protein identification; histological staining; Supplementary results Suppl. Figure 1. Full average spectrum spatial metabolomics and lipidomics; Suppl. Figure 2. SNU-601 induced tumor characterization via histology and molecular footprint clustering; Suppl. Figure 3. Spatial lipidomic analysis of SNU-601 induced tumor tissue; Suppl. Figure 4. Spatial metabolomics analysis of SNU-601 induced tumor tissue; Suppl. Figure 5. Untargeted proteomics analysis of SNU-601 induced tumortissue; Suppl. Figure 6. Specificity of ProteinTech (14681-1-AP) Rabbit polyclonalantibody against ASNS validated by chromogenic immunohistochemistry; Suppl. Figure 7. PCMT labeling confirmation; Suppl. Table 1. Identified lipids in the MALDI-MSI data set using lipidomicsLC-MS/MS data; Suppl. Table 2. Peptide identification using the MALDI-MSI and LC-MS/MSdata set; Suppl. Table 3. Significantly altered peptides in tumor samples 4 days after they received Asparlas; Suppl. Table 4. Significantly altered peptides in tumor samples 2 h received Asparlas (PDF)
