# Supplementary material for: Application of a Multiomics Imaging Workflow to Explore Asparlas Treatment in Solid Tumors
Source: Anal Chem. 2025 Jun 11;97(24):12736–45. doi: 10.1021/acs.analchem.5c01503 (PMC12199225; doi:10.1021/acs.analchem.5c01503)
Supplement: Supplementary file 1 [file ac5c01503_si_002.pdf]

Application of a multi-omics imaging workflow to explore Asparlas treatment in solid tumors

Laura van der Vloet [1], Ronny Mohren [1], Christophe Bouillod [2], Ron M.A. Heeren [1], Michiel Vandenbosch [1*±], and Pierre Barbier Saint Hilaire [2±]

[1] The Maastricht MultiModal Molecular Imaging (M4I) institute, Division of Imaging Mass Spectrometry (IMS), Maastricht University, 6229 ER Maastricht, The Netherlands

[2] Institut de Recherche et Développement SERVIER Paris-Saclay, 22 route 128, 91190 Gif-sur-Yvette, France

± These authors contributed equally to the manuscript.

Authors:
Laura van der Vloet - *The Maastricht MultiModal Molecular Imaging (M4I) institute, Division of Imaging Mass Spectrometry (IMS), Maastricht University, 6229 ER Maastricht, The Netherlands*
laura.vandervloet@maastrichtuniversity.nl
https://orcid.org/0000-0003-2478-8675

Ronny Mohren - *The Maastricht MultiModal Molecular Imaging (M4I) institute, Division of Imaging Mass Spectrometry (IMS), Maastricht University, 6229 ER Maastricht, The Netherlands*
r.mohren@maastrichtuniversity.nl
https://orcid.org/0000-0001-6673-5542

Christophe Bouillod - *Institut de Recherche et Développement SERVIER Paris-Saclay, 22 route 128, 91190 Gif-sur-Yvette, France*
christophe.bouillod@servier.com

Prof. Ron M.A. Heeren - *The Maastricht MultiModal Molecular Imaging (M4I) institute, Division of Imaging Mass Spectrometry (IMS), Maastricht University, 6229 ER Maastricht, The Netherlands*
r.heeren@maastrichtuniversity.nl
https://orcid.org/0000-0002-6533-7179

Michiel Vandenbosch - *The Maastricht MultiModal Molecular Imaging (M4I) institute, Division of Imaging Mass Spectrometry (IMS), Maastricht University, 6229 ER Maastricht, The Netherlands*
m.vandenbosch@maastrichtuniversity.nl
https://orcid.org/0000-0002-0427-416X

Pierre Barbier Saint Hilaire - *Institut de Recherche et Développement SERVIER Paris-Saclay, 22 route 128, 91190 Gif-sur-Yvette, France*
pierre.barbier-saint-hilaire@servier.com
https://orcid.org/0000-0002-3365-5316

Table of contents: Supplementary methods and materials

Lipid extraction of whole tumor tissue 2
LC-MS/MS lipidomics analysis 2
MALDI-IHC sample preparation 2
PC-MT labeling confirmation 2
Proteomics sample preparation 3
LC-MS/MS proteomics analysis and protein identification 3
Histological staining 4

Table of contents: Supplementary results

Suppl. Figure 1. Full average spectrum spatial metabolomics and lipidomics 5
Suppl. Figure 2. SNU-601 induced tumor characterization via histology and molecular footprint clustering 6
Suppl. Figure 3. Spatial lipidomic analysis of SNU-601 induced tumor tissue 7
Suppl. Figure 4. Spatial metabolomics analysis of SNU-601 induced tumor tissue 9
Suppl. Figure 5. Untargeted proteomics analysis of SNU-601 induced tumor tissue 10
Suppl. Figure 6. Specificity of ProteinTech (14681-1-AP) Rabbit polyclonal antibody against ASNS validated by chromogenic Immunohistochemistry 10
Suppl. Figure 7. PCMT labeling confirmation 11

Suppl. Table 1. Identified lipids in the MALDI-MSI data set using lipidomics LC-MS/MS data 12
Suppl. Table 2. Peptide identification using the MALDI-MSI and LC-MS/MS data set 13
Suppl. Table 3. Significantly altered peptides in tumor samples 4 days after they received Asparlas 14
Suppl. Table 4. Significantly altered peptides in tumor samples 2 hours after they received Asparlas 14

Supplementary methods and materials

Lipid extraction of whole tumor tissue

Lipids were extracted from thinly sectioned (5 µm) tumor tissue. Tissue sections were collected in 2.0 mL Eppendorf tubes that contained 375 µL methanol and vortexed for 10 sec. Next, 1250 µL MTBE was added to methanol suspension and was vortexed for 10 sec, followed by an incubation of 1 h at RT, and at 500 RPM. Consecutively, 350 µL MilliQ water was added, followed by vortexing for 10 sec, and was incubated for 10 min at RT. The suspension was then centrifuged for 10 min at 1000 *g* at RT. The upper non-polar liquid phase was collected into a new Eppendorf tube. The lower liquid phase was re-extracted with 600 µL MTBE, 180 µL methanol, and 150 µL MilliQ water. The mixture was vortexed for 1 min, followed by centrifuging for 10 min at 1000 *g* at RT. Lipids were collected from the upper layer and combined with the non-polar liquid phase. The resulting lipid mixture was concentrated in a SpeedVac and resuspended in acetonitrile/isopropanol (1:1 ratio).

LC-MS/MS lipidomics analysis

Lipid analysis was performed on a Thermo Scientific (Dionex) Ultimate 3000 Rapid Separation UHPLC system with a Thermo Scientific Hypersil Gold C18 analytical column (10cm, ID 2.1 mm, 1.9 µM). Separation was established by a 25 minute gradient starting from 68% mobile phase A (ACN:H2O 60:40 (v/v), 10 mM AF) to 97% mobile phase B (IPA:ACN 90:10 (v/v), 10 mM AF) at a flow rate of 0.25 ml/min. The UHPLC system was coupled to a high-mass resolution Orbitrap Ms Q-Exactive HF (Thermo Scientific) with a nano electrospray Flex ion source (Proxeon, Thermo Scientific). The spectrometer was programmed to run in data-dependent acquisition (DDA) mode, in positive ionization mode. Full MS scans were between 200 – 1,450 m/z at resolution of 60,000 followed by MS/MS scans of the top 8 most intense ions at a resolution of 30,000. The lipid species were subsequently assigned using MS1 and MS2 spectra acquired from DDA measurements in Lipostar2 version 2.1.7. Lipid identifications for MALDI-MSI were assigned by linking MS1 precursor ions found in the MALDI-MSI measurements to the MS1 + MS2 *m/z* values found in the LC-MS/MS measurements, using the LIPID MAPS database (3- and 4-star rating, Molecular Horizon, Bettona, PG, Italy).

MALDI-IHC sample preparation

SNU-601 induced tumor tissue sections were fixated by incubating in 1% PFA for 30 min, followed by a series of washing steps with PBS (1 x 10 min), acetone (2 x 3 min), and Carnoy's solution (1 x 3 min). This was followed by re-hydration, merging the slides in 100% EtOH (2 x 2 min), 95% EtOH (1 x 3 min), 70% EtOH (1 x 3 min), and 50% EtOH (1 x 3 min). The slides were washed with TBS for 10 min, and antigen retrieval using an alkaline buffer (pH = 9.0), was performed using the Retriever 2100 (Aptum Biologics Ltd, Rownhams, U.K.) for 20 min at 121°C. The slides were washed again with TBS for 10 min. Next, the tissue sections were incubated with blocking buffer for 1 h at RT. The blocking buffer was discarded, and the slides were incubated with the PC-MT labeled ASNS antibody (2 µg/mL in blocking buffer) overnight at 4°C. The slides were washed with TBS (3 x 5 min), ABC (1 x 10 sec), and ABC (3 x 2 min). The slides were completely dried in the desiccator for 1.5 h. The PC-MT antibody probe was photocleaved by illumination of UV light at 365 nm with a Phrozen UV curing lamp for 5 min (3 mW/cm^2) to achieve maximum photocleavage.

PC-MT labeling confirmation

Undiluted stock of Miralys Probes was transported to a 0.5 mL clear, thin-walled PCR type polypropylene tube. The Miralys probes were photocleaved by illumination of UV light at 365 nm with a Phrozen UV curing lamp for 5 min (3 mW/cm^2) to achieve maximum photocleavage. Meanwhile, 1x CHCA matrix was prepared by preparing 10 mg/mL CHCA

in TA30 solvent (3 ACN : 7 MS-water + 0.1% TFA). The solution was spinned down and the Miralys probe solution was diluted with spiked 1x CHCA matrix to a concentration of 10 µg/mL and 1 µg/mL. Of each solution, 1 µL was spotted on a ground steel 384-well MALDI plate and dried in a desiccator box for 5 min. MALDI-MSI spectra were acquired on a timstof fleX instrument (Bruker Daltonics GmbH, Bremen, Germany) in positive ionization mode at a pixel size of 50 x 50 µm. The laser frequency was set to 5,000 Hz, and 100 shots were accumulated at each pixel. Spectra were exported to Compas DataAnalysis and Mmass for further analysis.

Proteomics sample preparation

Fresh frozen SNU-601 induced tumor tissue sections (5 µm thickness) were collected in 1.5 mL Eppendorf. Containing 50 mM ABC buffer and were shortly centrifuged at 15,000 *g* to collect the tissue at the bottom of the tube. Next, 2,2 µL of 0.1% RapiGest was added to the sample and incubated for 10 min at RT, shaking at 800 rpm. Subsequently, samples underwent reduction by the addition of DTT (200 mM in 50 mM ABC for final [DTT] = 10 mM), which was incubated for 40 min at 800 rpm and 56°C. Next, samples underwent alkylation by the addition of IAM (400 mM in 50 mM ABC for final [IAM] = 20 mM), followed incubation for 10 min at 800 rpm and RT. Lastly, DTT (final [DTT] = 10 mM) was added and incubated for 10 min at 800 rpm and RT. For protein digestion, trypsin (final v/v = 15 µg/mL) was added prior overnight incubation for 16 h at 37°C and 800 rpm. After incubation, trypsin (final v/v = 5 µg/mL) and ACN (final [ACN] = 80%) were added to the samples, followed by a 3 h incubation at 800 rpm and 37°C. After incubation, TFA (final [TFA] = 0.5%) was added, and the sample was incubated for 45 min at 37°C and 800 rpm. Finally, the samples were centrifuged at 15,000 *g* for 15 min at 4°C. The resulting supernatant were collected in a new Eppendorf tube and concentrated in a SpeedVac and resuspended in 2% ACN and 0.05% TFA in HPLC graded water. Protein samples were stored at -20°C until further use.

LC-MS/MS proteomics analysis and protein identification

Fresh frozen SNU-601 induced tumor tissue sections (5 µm thickness) were collected in 1.5 mL Eppendorf tubes. The samples were prepared for tandem LC-MS proteomics analysis using a standard protein digestion protocol. Peptide separation was performed on a Thermo Scientific (Dionex) Ultimate 3000 Rapid Separation UHPLC system equipped with a Thermo Scientific Acclaim PepMap C18 analytical column (15 cm, ID 75 µm, 3 µm). Peptide samples were first desalted on an online installed C18 trapping column. After desalting, peptides were separated on the analytical column with a 110 minute gradient from 4% to 32% Acetonitrile (ACN) with 0.1% FA at 300 nL/min flow rate. The UHPLC system was coupled to a Q Exactive HF mass spectrometer (Thermo Scientific). DDA settings were as follows. Full MS scan between 250 – 1,250 m/z at resolution of 120,000 followed by MS/MS scans of the top 15 most intense ions at a resolution of 15,000.

Protein identification was performed in Proteome Discoverer software version 2.2 (Thermo Scientific) in which the raw files were processed using the search engine Sequest with the Uniprot protein database *Homo Sapiens* (TaxID 9606). The following settings were used for the protein database search: trypsin was used as the enzyme with a maximum of two missed cleavage sites and a minimum peptide length of six amino acids. The mass window for the precursor was set at 350 – 5,000 Da. The mass tolerance of the precursor and fragment were 10 ppm and 0.02 Da, respectively. Acetylation on the n-terminus and methionine oxidation were used as dynamic modifications, and carbomidomethylation was used as static modification. A strict false discovery rate (FDR) of 0.01 was used to estimate the confidence of the identification.

Histological staining

A consecutive slide after spatial metabolomics containing fresh frozen subcutaneous tumor tissue sections were first dried in a desiccator. Next, the slides were rehydrated by submerging the slides in an EtOH series: 3 min 100% EtOH, 2 x 3 min 96% EtOH, 2 x 3 min 70% EtOH, and 3 min MilliQ. The slides were then incubated with hematoxylin for 3 min, followed by rinsing the slides with tap water until the hematoxylin (Merck) residues were removed from the slide. Consequently, the slides were incubated with eosin (Klinipath) for 30 sec and rinsed with tap water. Lastly, the slides were incubated in 100% EtOH for 1 min, and 30 sec in Xylol. The slides were covered with Entallan and covered with a coverslip. The Aperio CS2 scanner (Leica Microsystems) was used for whole slide scanning and digitalization at a 20x magnification.

Supplementary results

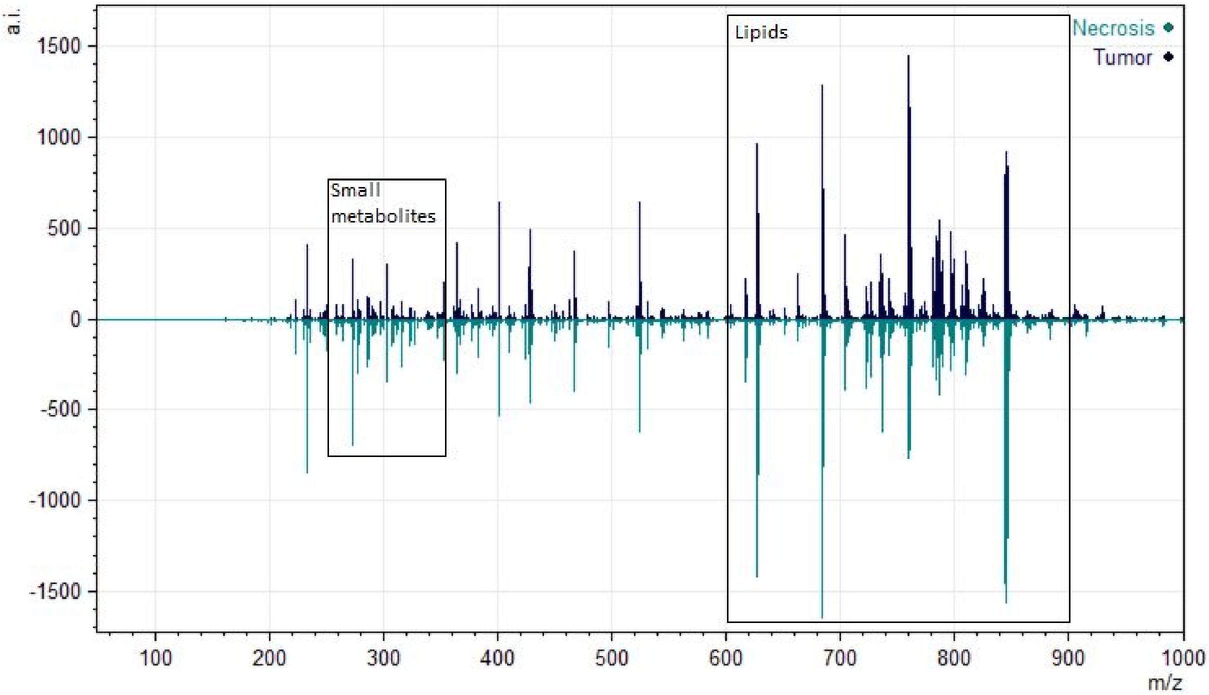

Suppl. Figure 1. Full average spectrum spatial metabolomics and lipidomics. *In dark blue, the full spectrum of the tumor regions is presented, as where in green the full spectrum of the necrotic regions is presented. The small metabolite (after CA derivatization) and lipid range are highlighted within a box. Data is root mean square normalized.*

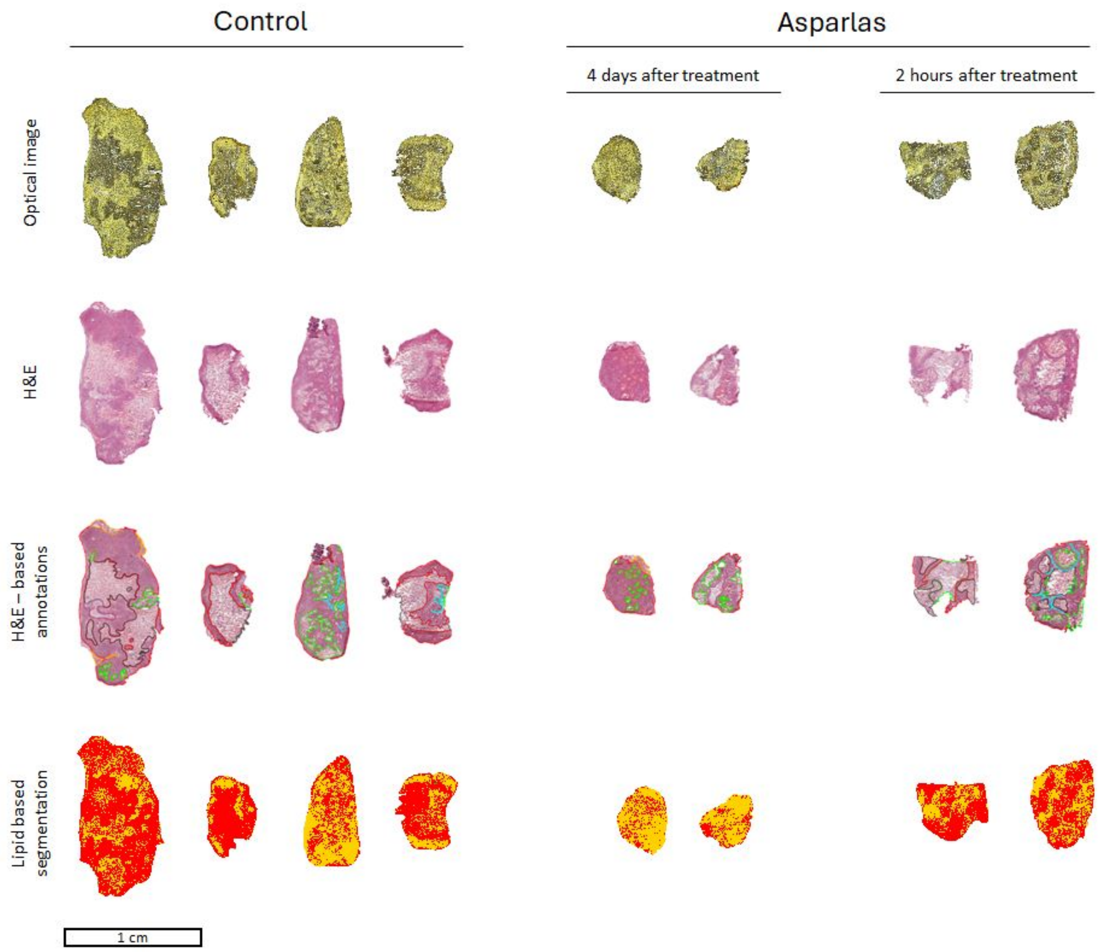

Suppl. Figure 2. SNU-601 induced tumor characterization via histology and molecular footprint clustering. *CA derivatization was applied prior MALDI-MSI analysis to enable the simultaneously detection of metabolites (e.g. amino acids) and lipids. CA derivatization caused a yellow stain on tissue, for which histology was performed on a consecutive slide, followed by annotations. H&E-based annotations were categorized in red = tumor, black = necrosis, green = exudate, blue = stroma, yellow = immune cells. Based on acquired spatial lipidomic data, segmentation was performed, which resulted in two main clusters. Cluster 1 in yellow represents tumor tissue, and cluster 2 in red represents necrotic and stroma tissue.*

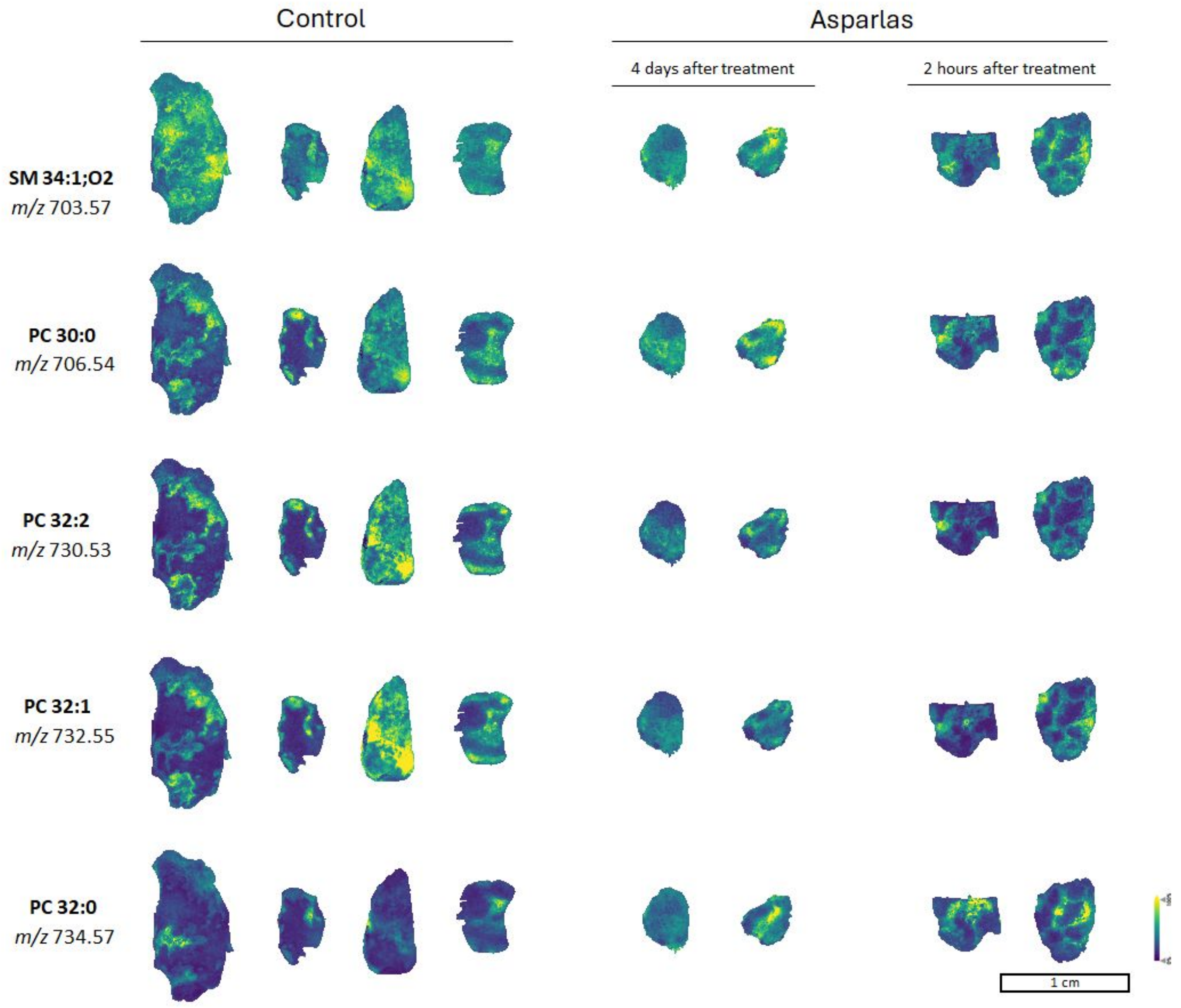
Control
Asparlas
4 days after treatment
2 hours after treatment
SM 34:1;O2
m/z 703.57
PC 30:0
m/z 706.54
PC 32:2
m/z 730.53
PC 32:1
m/z 732.55
PC 32:0
m/z 734.57
1 cm

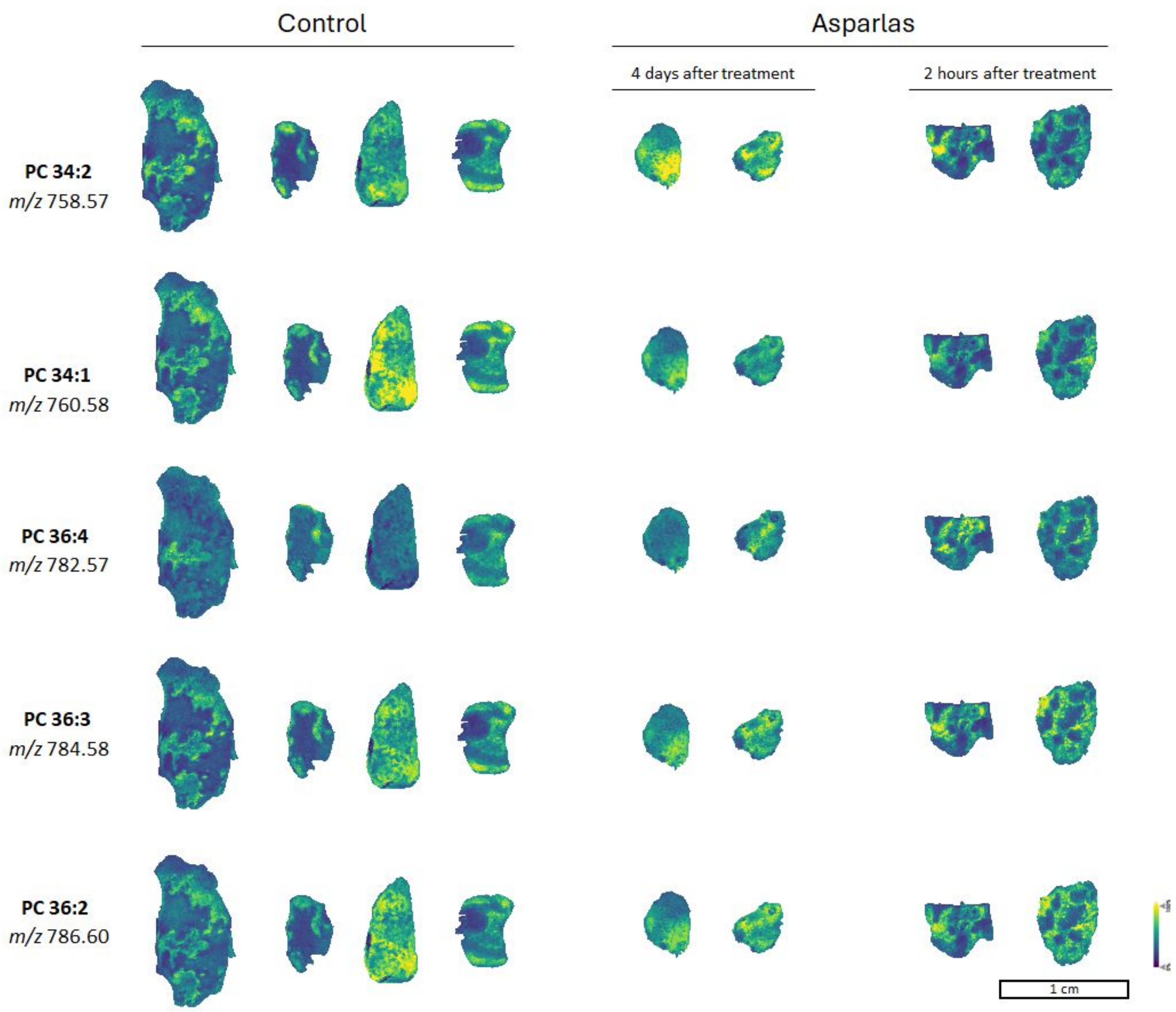

Suppl. Figure 3. Spatial lipidomic analysis of SNU-601 induced tumor tissue. *Lipids that are responsible for the molecular clustering in suppl. Figure 2. Ion images are root mean square normalized.*

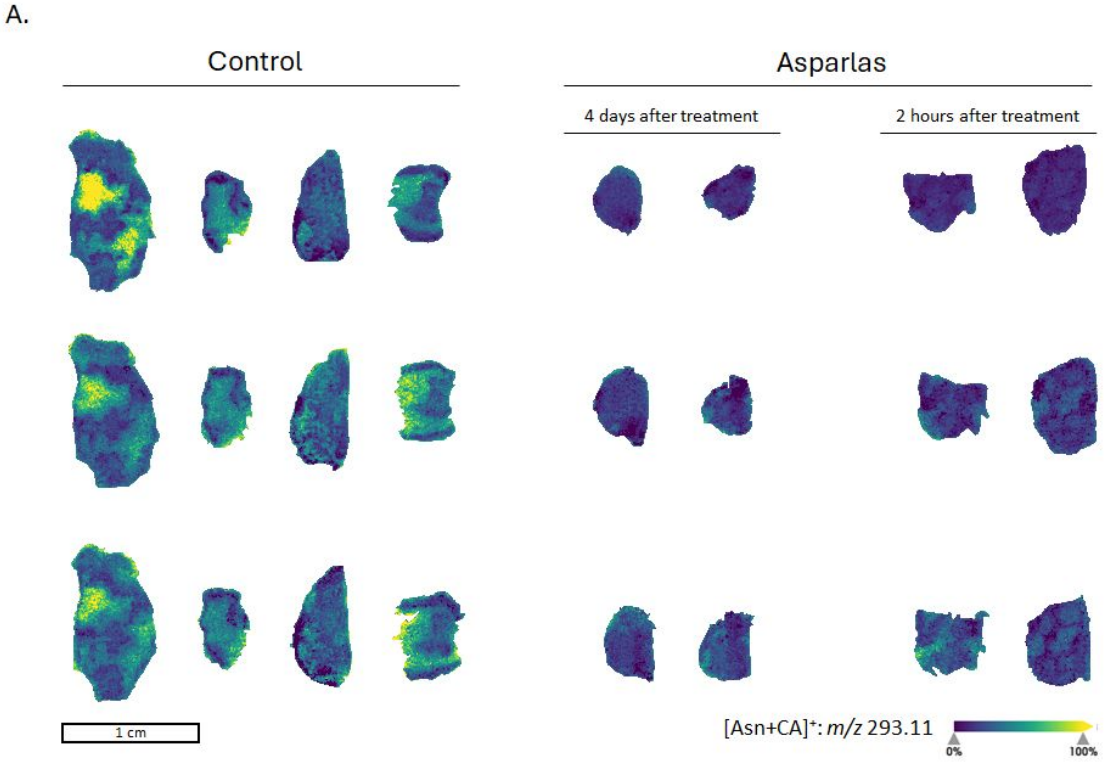

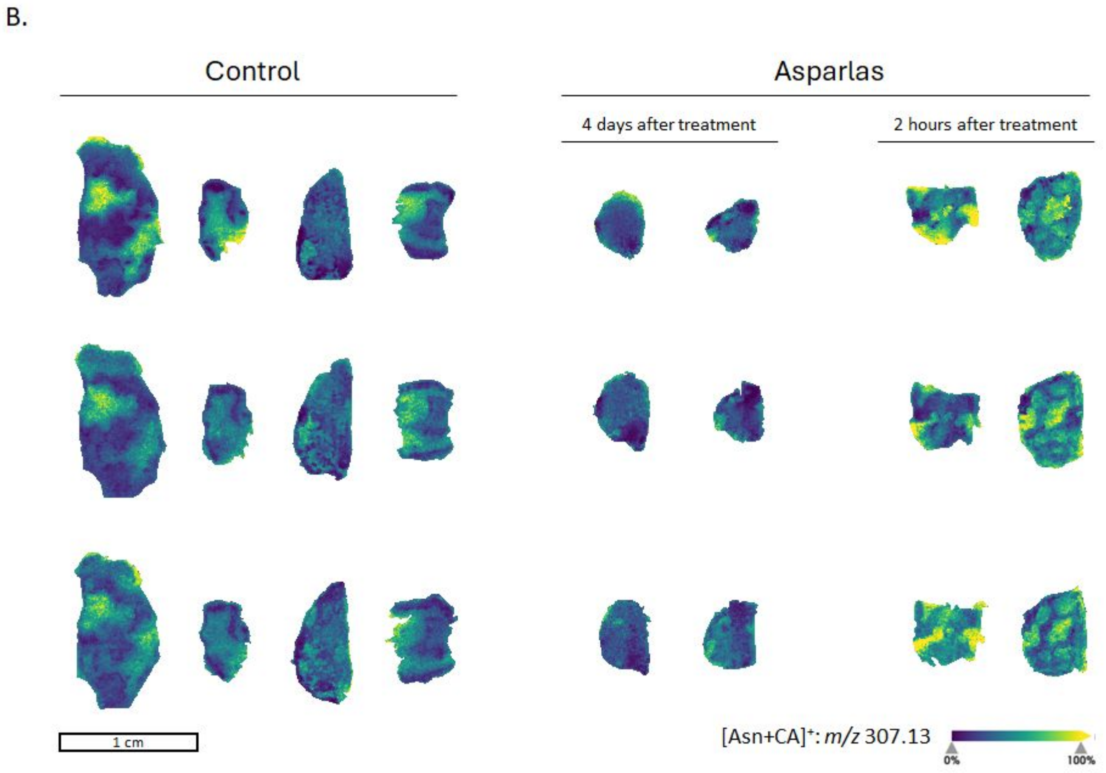

Suppl. Figure 4. Spatial metabolomics analysis of SNU-601 induced tumor tissue. ***A.*** *Asn expression in control and Asparlas treated tissues.* ***B.*** *Gln expression in control and Asparlas treated tissues. Ion images are root mean square normalized.*

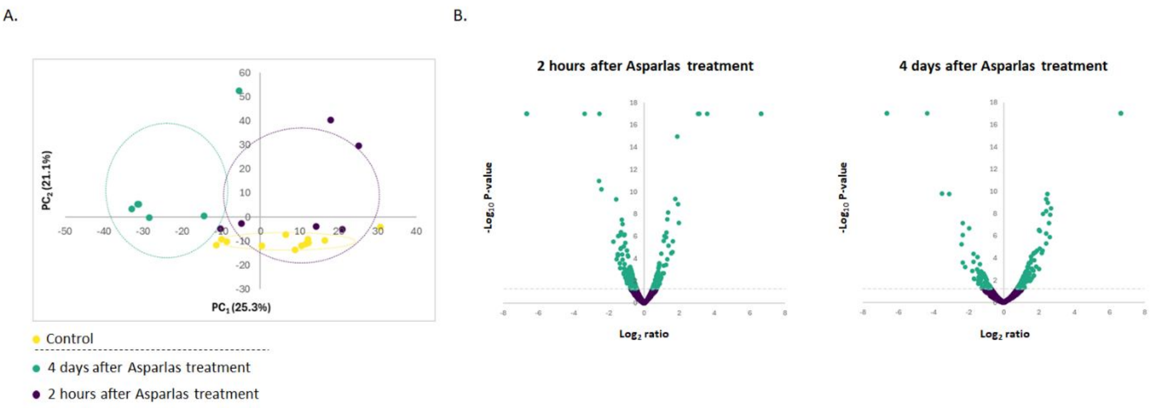

Suppl. Figure 5. Untargeted proteomics analysis of SNU-601 induced tumor tissue. A. *A 2D PCA plot of the LC-MS proteomic data set, with the axes representing PC_1 and PC2. 95% confidence ellipses are presented for each experimental group.* ***B.*** *Volcano plot of the 1974 identified proteins from the LC-MS data set. In green all the significantly altered proteins. False discovery rate < 1% and significance is shown as: *p < 0.05. Proteins upregulated in Asparlas treated groups are presented on the left side of the x-axis. Down-regulated proteins in the Asparlas treated groups are presented on the right side of the x-axis.*

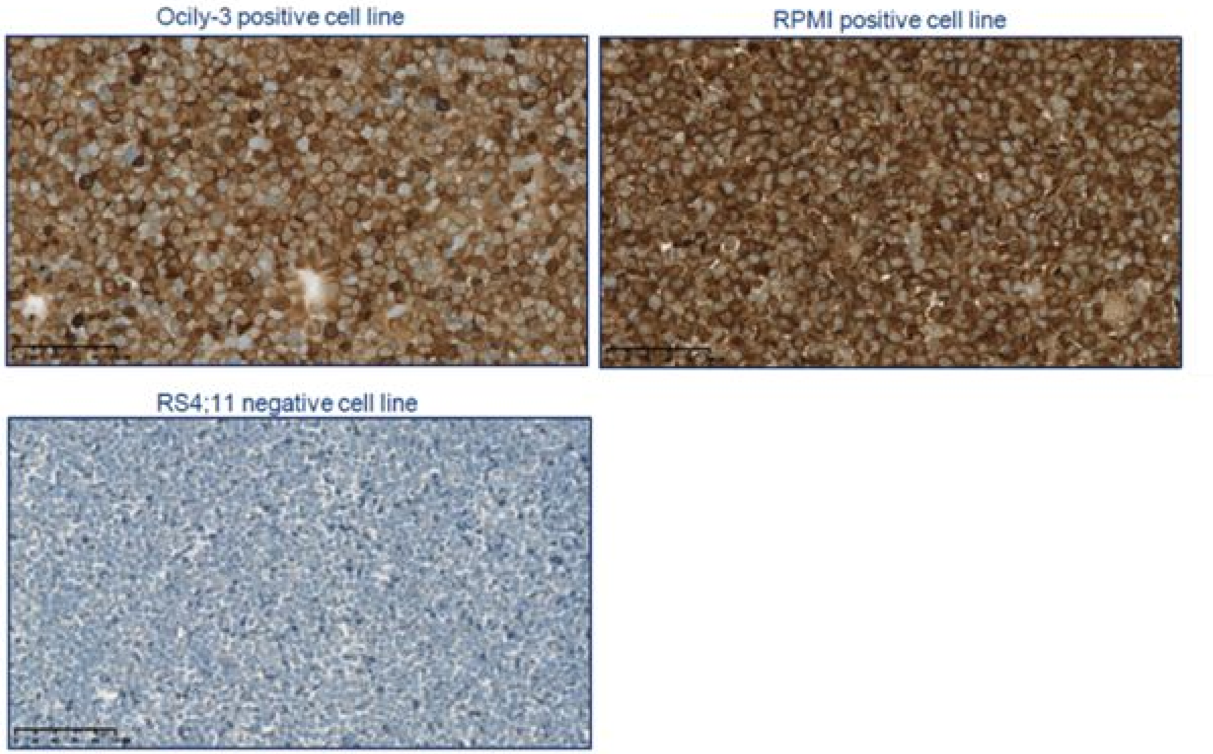

Suppl. Figure 6. Specificity of ProteinTech (14681-1-AP) Rabbit polyclonal antibody against ASNS validated by chromogenic Immunohistochemistry. *Positive control cell lines: OCILY-3 and RPMI-8226; Negative control cell line: RS4-11.*

A.

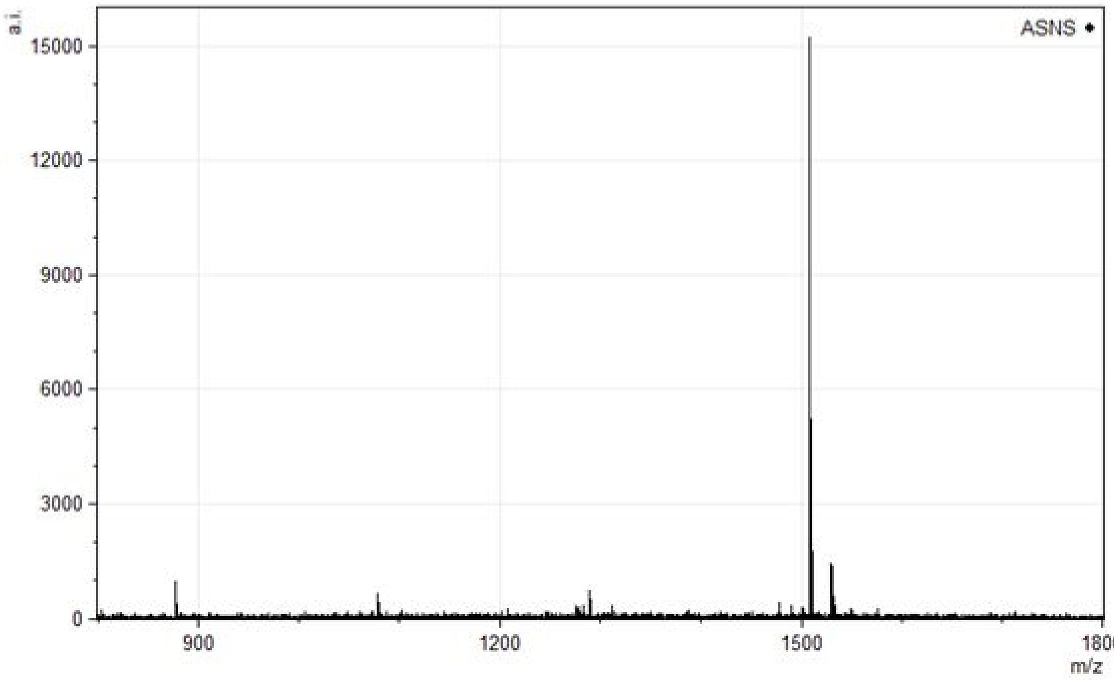

B.

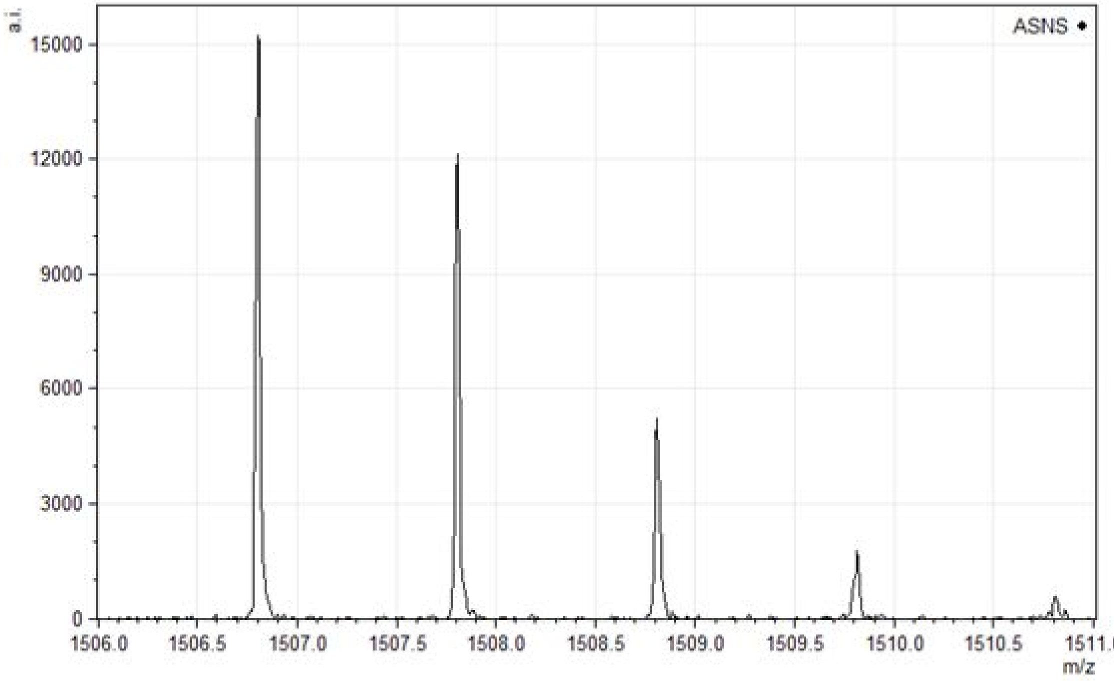

Suppl. Figure 7. PCMT labeling confirmation. A. *Full average spectrum of the PC-MT labeled antibody spiked in CHCA.* ***B.*** *Average spectrum zoomed in on the mass of the PC-MT (1506.76 m/z).*

Suppl. Table 1. Identified lipids in the MALDI-MSI data set using lipidomics LC-MS/MS data.

MALDI-MSI (m/z)	LC-MS/MS (m/z)	Theoretical value (m/z)	Lipid ID	Mass error (ppm) - LC-MS/MS	Mass error (ppm) - MALDI MSI	Adduct	AUC
520.34	520.3408	520.3398	LPC 18:2	1.92	-1.15	$[M+H]^+$	0.564
522.35	522.3566	522.3554	LPC 18:1	2.30	-2.30	$[M+H]^+$	0.576
524.37	524.3720	524.3711	LPC 18:0	1.72	-3.81	$[M+H]^+$	0.445
536.50	536.5050	536.5037	Cer 34:2;O2	2.42	-2.61	$[M+H]^+$	0.454
538.52	538.5207	538.5194	Cer 34:1;O2	2.41	-2.60	$[M+H]^+$	0.527
540.53	540.5363	540.535	Cer 34:0;O2	2.41	-3.52	$[M+H]^+$	0.555
544.34	544.3410	544.3398	LPC 20:4	2.20	2.20	$[M+H]^+$	0.573
620.60	620.5983	620.5976	Cer 40:2;O2	1.13	-2.09	$[M+H]^+$	0.476
622.61	622.6146	622.6133	Cer 40:1;O2	2.09	0.16	$[M+H]^+$	0.498
624.63	624.6297	624.6289	Cer 40:0;O2	1.28	-0.32	$[M+H]^+$	0.534
648.63	648.6300	648.6289	Cer 42:2;O2	1.70	-0.15	$[M+H]^+$	0.447
650.64	650.6460	650.6446	Cer 42:1;O2	2.15	-2.92	$[M+H]^+$	0.484
676.66	676.6614	676.6602	Cer 44:2;O2	1.77	-3.55	$[M+H]^+$	0.486
701.56	701.5640	701.5592	SM 34:2;O2	6.84	-4.70	$[M+H]^+$	0.239
703.57	703.5761	703.5749	SM 34:1;O2	1.71	-3.70	$[M+H]^+$	0.213
705.59	705.5920	705.5905	SM 34:0;O2	2.13	-4.39	$[M+H]^+$	0.294
706.54	706.5396	706.5381	PC 30:0	2.12	-1.56	$[M+H]^+$	0.134
716.52	716.5240	716.5225	PE 34:2	2.09	2.09	$[M+H]^+$	0.328
718.54	718.5395	718.5381	PE 34:1	1.95	-3.20	$[M+H]^+$	0.278
718.57	718.5761	718.5745	PC O-32:1	2.23	-3.76	$[M+H]^+$	0.302
719.57	719.5763	719.5738	CE 22:6	3.47	1.53	[M+Na]+	0.279
724.52	724.5274	724.5276	PE O-36:5	-0.28	-4.28	$[M+H]^+$	0.344
725.55	725.5580	725.5568	SM 34:1;O2	1.65	-3.72	$[M+Na]^+$	0.490
730.54	730.5397	730.5381	PC 32:2	2.19	-0.82	$[M+H]^+$	0.175
732.55	732.5552	732.5538	PC 32:1	1.91	-1.23	$[M+H]^+$	0.171
734.57	734.5709	734.5694	PC 32:0	2.04	-3.95	$[M+H]^+$	0.187
740.52	740.5234	740.5225	PE 36:4	1.22	-5.40	$[M+H]^+$	0.351
744.56	744.5560	744.5538	PE 36:2	2.95	3.22	$[M+H]^+$	0.242
746.60	746.6075	746.6058	PC O-34:1	2.28	-1.74	$[M+H]^+$	0.349
748.53	748.5294	748.5276	PE O-38:7	2.40	-2.54	$[M+H]^+$	0.346
750.54	750.5442	750.5408	PE O-36:3	4.53	-3.46	$[M+Na]^+$	0.398
752.56	752.5622	752.5589	PE O-38:5	4.39	-3.72	$[M+H]^+$	0.344
754.53	754.5373	754.5357	PC 32:1	2.12	-2.12	$[M+Na]^+$	0.344
756.55	756.5520	756.5514	PC 32:0	0.79	0.00	$[M+Na]^+$	0.242
758.57	758.5718	758.5694	PC 34:2	3.16	-3.82	$[M+H]^+$	0.126
760.58	760.5867	760.5851	PC 34:1	2.10	-2.50	$[M+H]^+$	0.142
766.54	766.5357	766.5357	PE 38:5	0.00	-0.78	$[M+H]^+$	0.343
768.59	768.5918	768.5902	PC O-34:1	2.08	-2.47	$[M+Na]^+$	0.308
770.51	770.5110	770.5095	PE O-38:7	1.95	-1.43	$[M+Na]^+$	0.322
770.61	770.6076	770.6058	PC O-36:3	2.34	2.34	$[M+H]^+$	0.341
780.55	780.5552	780.5538	PC 36:5	1.79	-3.20	$[M+H]^+$	0.354
782.57	782.5711	782.5694	PC 36:4	2.17	-2.04	$[M+H]^+$	0.199
784.58	784.5868	784.5851	PC 36:3	2.17	-4.59	$[M+H]^+$	0.125
786.60	786.6038	786.6007	PC 36:2	3.94	-0.25	$[M+H]^+$	0.136
788.61	788.6124	788.6164	PC 36:1	-5.07	-4.56	$[M+H]^+$	0.219
792.59	792.5925	792.5902	PC O-38:6	2.90	-5.80	$[M+H]^+$	0.290
794.57	794.5710	794.5694	PE 40:5	2.01	0.13	$[M+H]^+$	0.338
794.60	794.6070	794.6058	PC O-38:5	1.51	-5.66	$[M+H]^+$	0.276
798.54	798.5425	798.5408	PE O-40:7	2.13	-5.64	$[M+Na]^+$	0.354
804.55	804.5529	804.5514	PC 36:4	1.86	-4.35	$[M+Na]^+$	0.335
806.57	806.5711	806.5694	PC 38:6	2.11	-4.46	$[M+H]^+$	0.225
808.58	808.5843	808.5827	PC 36:2	1.98	-2.47	$[M+Na]^+$	0.284
810.60	810.6024	810.6007	PC 38:4	2.10	-5.06	$[M+H]^+$	0.230
820.62	820.6213	820.6215	PC O-40:6	-0.24	-6.58	$[M+H]^+$	0.336
828.55	828.5532	828.5514	PC 38:6	2.17	1.57	$[M+Na]^+$	0.388
830.57	830.5713	830.5694	PC 40:8	2.29	2.29	$[M+H]^+$	0.318
832.58	832.5869	832.5851	PC 38:4	2.16	-5.04	$[M+Na]^+$	0.325
834.59	834.6012	834.6007	PC 40:6	0.60	-7.91	$[M+H]^+$	0.245
836.61	836.6190	836.6164	PC 40:5	3.11	-5.26	$[M+H]^+$	0.249

Suppl. Table 2. Peptide identification using the MALDI-MSI and LC-MS/MS data set.

Protein ID	Protein description	Sequence	Monoisotopic mass	Theoretical [M+H]+	Experimental LC-MS [M+H]+	Experimental MALDI-MSI [M+H]+
P68133; P68032; P63261	Actin	AVFPSIVGRPR	1197.69	1198.69	1198.71	1198.72
		AGFAGDDAPR	975.43	976.44	976.45	976.46
		HQGVMVGMGQK	1170.55	1171.56	1171.57	1171.58
P17301	Integrin alpha-2	AIASIPTER	956.52	957.53	957.54	957.55
		SVACDVGYPALK	1221.60	1222.61	1279.64	1279.66
		FVQGLDIGPTK	1173.63	1174.64	1174.65	1174.65
Q99715	Collagen alpha-1(XII) chain	VLVVVTDGR	956.55	957.56	957.57	957.55
		IGVLITDGK	914.53	915.54	915.55	915.54
		GGNTMTGDAIDYLVK	1553.73	1554.74	1554.75	1554.77
P21333; O75369; Q14315	Filamin	LLGWIQNK	970.55	971.56	971.57	971.57
		SPFEVQVGPEAGMQK	1602.76	1603.77	1603.78	1603.79
		DAGYGGISLAVEGPSK	1519.74	1520.75	1520.76	1520.77
Q05707	Collagen alpha-1(XIV) chain	VIVVITDGR	970.57	971.58	971.59	971.57
		IGILITDGK	928.55	929.56	929.57	929.56
		-	-	-	-	-
Q6S8J3	POTE ankyrin domain family member E	AVFPSIVGRPR	1197.69	1198.69	1198.71	1198.72
		AGFAGDDAPR	975.43	976.44	976.45	976.46
		SYELPDGQVITIGNER	1789.87	1790.88	1790.89	1790.91
P84077	ADP-ribosylation factor 1	MLAEDELR	975.46	976.47	976.48	976.46
		-	-	-	-	-
		-	-	-	-	-
Q99873	Protein arginine N-methyltransferase	VIGIECSSISDYAVK	1582.78	1583.79	1640.82	1640.82
		ATLYVTAIEDR	1250.64	1251.65	1251.66	1251.65
		WLAPDGLIFPDR	1398.72	1399.73	1399.74	1399.78
P04075	Fructose-bisphosphate aldolase A	LQSIGTENTEENRR	1645.79	1646.80	1646.81	1646.82
		ADDGRPFPQVIK	1341.69	1342.70	1342.71	1342.72
		GVVPLAGTNGETTTQGLDGL	2271.12	2272.13	2272.14	2272.17
P17858	ATP dependent 6-phosphofructokinase	SEWGSLLEELVAEGK	1645.81	1646.82	1646.83	1646.82
		-	-	-	-	-
		-	-	-	-	-
P15924	Desmoplakin	NATILELR	928.52	929.53	929.54	929.56
		QLQNIIQATSR	1270.69	1271.70	1271.71	1271.70
		GFFDPNTEENLTYLQLK	2027.97	2028.98	2028.99	2029.02
P12429	Annexin 3A	ALLTLADGR	928.52	929.53	929.54	929.56
		-	-	-	-	-
		-	-	-	-	-
P55327	Tumor protein D52	LGINSLQELK	1113.63	1114.64	1114.65	1114.64
		-	-	-	-	-
		-	-	-	-	-
P52272	Heterogenous nuclear ribonucleoprotein M	INEILSNALK	1113.63	1114.64	1114.65	1114.64
		-	-	-	-	-
		-	-	-	-	-
Q9Y3B4	Splicing factor 3B subunit 6	ITAEEMYDIFGK	1415.65	1416.66	1416.67	1416.67
		-	-	-	-	-
		-	-	-	-	-
Q15149	Plectin	LEDLLQDAQDEK	1415.67	1416.67	1416.69	1416.67
		GDECQLVGPAQPSHWK	1750.80	1751.81	1808.84	1808.92
		AYSDPSTGEPATYGELQQR	2068.92	2069.93	2069.94	2069.97
Q9NQP4	Prefoldin subunit 4	NLQEEIDALESR	1415.68	1416.69	1416.7	1416.67
		-	-	-	-	-
		-	-	-	-	-
P13535; P12882; P12883; Q9UKX2; Q9Y623	Myosin	AITDAAMMAEELKK	1520.75	1521.75	1521.77	1521.77
		SALAHALQSSR	1139.59	1140.60	1140.61	1140.61
		AAYLQNLNSADLLK	1532.81	1533.82	1533.83	1533.82
Q9NZM1	Myoferlin	NLVDPFVEVSFAGK	1520.78	1521.78	1521.79	1521.77
		ISVYDYDTFTR	1378.63	1379.64	1379.65	1379.64
		-	-	-	-	-
O75955	Flotillin-1	SQLIMQAEAEAASVR	1602.79	1603.80	1603.81	1603.79
		-	-	-	-	-
		-	-	-	-	-
P02751	Fibronectin	VTIMWTPPESAVTGYR	1806.89	1807.89	1807.9	1807.92
		GLAFTDVDVDSIK	1378.69	1379.69	1379.71	1379.71
		EATIPGHLNSYTIK	1542.79	1543.80	1543.81	1543.76
P23246	Splicing factor proline- and glutamine-rich	LFVGNLPADITEDEFK	1806.89	1807.90	1807.91	1807.92
		YGEPGEVFINK	1251.60	1252.61	1252.62	1252.65
		FGQGGAGPVGGQGPR	1340.65	1341.66	1341.67	1341.68
O00391	Sulfhydryl oxidase 1	IYMADLESALHYILR	1806.92	1807.93	1807.94	1807.92
		-	-	-	-	-
		-	-	-	-	-
P38646	Stress-70 protein, mitochondrial	SQVFSTAADGQTQVEIK	1807.88	1808.89	1808.9	1808.92
		EQQIVIQSSGGLSK	1472.77	1473.78	1473.79	1473.79
		-	-	-	-	-

Suppl. Table 3. Significantly altered peptides in tumor samples 4 days after they received Asparlas.

Protein ID	MALDI-MSI (m/z)	ASPARLAS dosed	AUC
Integrin alpha-2, Collagen alpha-1	957.55	↓	0.701
Filamin, Collagen Alpha-1	971.57	↓	0.703
ADP-ribosylation factor 1	976.46	↓	0.761
POTE ankyrin domain family member E, Actin	1198.72	↓	0.752
Myosin -1, 2, 4, 7, 8, myoferlin	1521.77	↓	0.712
protein arginine N-methyltransferase	1640.82	↓	0.738
fructose-bisphosphate aldolase A, ATP dependent 6-phosphofructokinase	1646.82	↓	0.713

Suppl. Table 4. Significantly altered peptides in tumor samples 2 hours after they received Asparlas.

Protein ID	MALDI-MSI (m/z)	ASPARLAS dosed	AUC
Tumor protein D52, Heterogenous nuclear ribonucleoprotein M	1114.64	↑	0.290
